# Single-cell RNAseq and longitudinal proteomic analysis of a novel semi-spontaneous urothelial cancer model reveals tumor cell heterogeneity and pretumoral urine protein alterations

**DOI:** 10.1371/journal.pone.0253178

**Published:** 2021-07-07

**Authors:** Iliana K. Kerzeli, Martin Lord, Milena Doroszko, Ramy Elgendy, Aikaterini Chourlia, Ivan Stepanek, Elinor Larsson, Luuk van Hooren, Sven Nelander, Per-Uno Malmstrom, Anca Dragomir, Ulrika Segersten, Sara M. Mangsbo

**Affiliations:** 1 Department of Pharmaceutical Biosciences, Science for Life Laboratory, Uppsala University, Uppsala, Sweden; 2 Department of Immunology, Genetics and Pathology, Science for Life Laboratory, Uppsala University, Uppsala, Sweden; 3 Department of Surgical Sciences, Uppsala University, Uppsala, Sweden; Centro Nacional de Investigaciones Oncologicas, SPAIN

## Abstract

Bladder cancer, one of the most prevalent malignancies worldwide, remains hard to classify due to a staggering molecular complexity. Despite a plethora of diagnostic tools and therapies, it is hard to outline the key steps leading up to the transition from high-risk non–muscle-invasive bladder cancer (NMIBC) to muscle-invasive bladder cancer (MIBC). Carcinogen-induced murine models can recapitulate urothelial carcinogenesis and natural anti-tumor immunity. Herein, we have developed and profiled a novel model of progressive NMIBC based on 10 weeks of OH-BBN exposure in hepatocyte growth factor/cyclin dependent kinase 4 (R24C) (Hgf-Cdk4^R24C^) mice. The profiling of the model was performed by histology grading, single cell transcriptomic and proteomic analysis, while the derivation of a tumorigenic cell line was validated and used to assess *in vivo* anti-tumor effects in response to immunotherapy. Established NMIBC was present in females at 10 weeks post OH-BBN exposure while neoplasia was not as advanced in male mice, however all mice progressed to MIBC. Single cell RNA sequencing analysis revealed an intratumoral heterogeneity also described in the human disease trajectory. Moreover, although immune activation biomarkers were elevated in urine during carcinogen exposure, anti-programmed cell death protein 1 (anti-PD1) monotherapy did not prevent tumor progression. Furthermore, anti-PD1 immunotherapy did not control the growth of subcutaneous tumors formed by the newly derived urothelial cancer cell line. However, treatment with CpG-oligodeoxynucleotides (ODN) significantly decreased tumor volume, but only in females. In conclusion, the molecular map of this novel preclinical model of bladder cancer provides an opportunity to further investigate pharmacological therapies ahead with regards to both targeted drugs and immunotherapies to improve the strategies of how we should tackle the heterogeneous tumor microenvironment in urothelial bladder cancer to improve responses rates in the clinic.

## Introduction

Bladder cancer holds the 10th position among the most common cancers worldwide [1]. Around 70% of patients are diagnosed with NMIBC out of which one fifth are stage T1. Due to limited predictive biomarkers of progression of T1 tumors, the majority of patients with high-risk T1 bladder tumors present recurrences, and almost half of those progress to MIBC, resulting in a 15% mortality within 10 years from diagnosis [2]. The treatment of NMIBC consists of transurethral resection of bladder tumors (TURBT), usually combined with a single intravesical administration of chemotherapy, or in high-risk cases, with additional treatment by Bacillus Calmette-Guerin (BCG) for up to three years [3,4].

Recently, a phase II clinical trial of anti-PD1 therapy for BCG resistant and recurrent tumors resulted in a complete response rate of 31% [5]. However, the high incidence of non-responders to immune checkpoint blockade therapy indicates the importance of studying immunotherapy resistance mechanisms and alternative treatments. Moreover, there is an altered disease distribution between genders. Generally, women have four times lower incidence of bladder cancer whilst presenting with a more advanced disease at diagnosis and higher disease-related morbidity than men [1,6–8]. While gender specific genomic alterations, such as the mutation in the KDM6A gene in women at the NMIBC stage have been revealed [9], the underlying cause of the gender disproportion is unknown. However, also the outcome of cancer immunotherapy is shown to be disproportionate between genders [10,11]. In mice, the immune landscape on baseline and upon immune activation also vary among males and females [12]. Hence, it is crucial to differentiate gender-biased molecular markers both in pre-clinical and clinical studies when assessing novel therapeutics.

So far, sex-focused studies on the outcome of BCG therapy have been hampered by the limited number of women in cohorts [13]. In line with that, pre-clinical therapeutic studies in murine models of bladder cancer with checkpoint inhibitors and immune modulation by TLR stimulation (BCG or CpG ODN) have been informative, but still lacked information of sex-specific effects [14–16]. Among pre-clinical models used to study urothelial cancer, are wild type animals exposed to OH-BBN [N-butyl-N-(4-hydroxybutyl) nitrosamine] [17], genetically engineered murine (GEM) models [18] or allografted tumorigenic cell lines such as MB49 (DMBA-*in vitro* transformed urothelial cells) [19] and MBT-2 (FANFT-induced bladder tumor in C3H/He mouse) [20]. The subtype and grade of OH-BBN induced tumors depend on genetic background, strain and sex [16,21]. In wild type C57BL/6 mice, prolonged exposure to OH-BBN (20 weeks) is required to induce MIBC that represents the molecular subtypes and mutational landscape found in humans [22–24]. However, commonly only male mice of the C57BL/6 strain are used in such studies, disregarding the sex-biased disease outcome and response to therapy [16,21,22]. On the other hand, most GEM models acquire urothelial hyperplasia and papillary non-progressive NMIBC instead of MIBC [25], and do not reflect the heterogeneous mutational landscape and gene expression profile of human tumors [16,22,23]. Previously, a GEM model that progressed from NMIBC to MIBC was induced via Cre/lox targeted deletion of *Tp53* and *Pten* in the urothelium [18,25,26]. Although such mutations are common in human bladder cancer, the small number of gene promoters found exclusively in the urothelium is an obstacle when generating models with urothelium-specific genetic modification via targeted gene transduction [18,27,28].

Urothelial bladder cancer as a disease has a strong prevalence of oncogenic driver mutations in combination with an immune infiltrated or “hot” pro-tumorigenic tumor microenvironment (TME), which suggest a need for a proper model where one can study a combination of small molecule targeting strategies along with immunotherapy approaches. In this study, we approached this by developing an OH-BBN induced urothelial bladder cancer model that combines two pro-oncogenic factors, overexpression on *Hgf* and an activating mutation in *Cdk4 (R24C)*. According to the Pathology Atlas of the Human Cancer Transcriptome high mRNA expression of HGF or CDK4 in urothelial bladder cancer correlates with a worse overall survival compared to patients with low expression of these genes (P-scores: HGF 0.021 and CDK4 0.016) [29]. Moreover, moderate protein expression for both genes is observed in urothelial cells as well as other cell types of the urinary bladder [29].

In urothelial bladder cancer, the alterations of the Hgf/c-met axis are commonly found in high-risk tumors [30–34] as well as in animal models [35,36]. The activating mutation R24C of *Cdk4* allows cells to overcome senescence and to acquire a proliferation phenotype by promoting the aberrant phosphorylation of Rb which inactivates its tumor suppressor function. The expression of the mutated *Cdk4 (R24C)* results in spontaneous development of epithelial tumors or sarcomas in mice at an older age (>8 months) or melanoma upon skin exposure to carcinogens [37–39]. Cell-cycle dysregulation of urothelial cancer in humans is commonly identified as either loss of function of RB [40–42] along with CDK4 upregulation [43–46] or genetic alterations of CDKN2A that usually hamper the function of its transcript p16 which is the inhibitor of CDK4 [47,48], herein modelled in mice by overactivation of *Cdk4* due to a point mutation [37,49,50]. Additionally, scattered mutations were introduced to the developing urothelial tumors by administration of the carcinogen OH-BBN [22].

The Hgf-Cdk4^R24C^ strain has been previously used to study melanoma-genesis upon exposure to carcinogenic stimuli [49,51], but is susceptible to spontaneous melanoma and to a lesser extent hepatocellular carcinoma development at an average age older than 8 months [52]. Moreover, this strain has previously been validated as potential model of spontaneous conjunctival or uveal melanoma, however there was no evidence of such spontaneously occurring pathology [53].

Herein, we subjected the animals to experiments of carcinogen induction or as healthy controls during the first 8 months of their life without observing high rates of spontaneous malignancy development, while using fully developed animals. Despite the presence of the genetic modifications in all cells, the carcinogen exposure in our study yielded bladder tumors that were histologically identified as urothelial carcinoma with squamous differentiation. The simultaneous overexpression of *Hgf* and increased activity of *Cdk4 (R24C)* led to accelerated progression of OH-BBN induced urothelial tumors compared to *Cdk4 (R24C)* alone. Using this novel model, we characterized it in comparison to commonly used models (OH-BBN exposed C57BL/6 mice and the syngeneic MB49 model) using transcriptomics, proteomics and assessed response to immunotherapy in both, male and female mice.

## Materials and methods

### Mice

C57BL/6(J) mice were purchased from Taconic M&B (Bomholt, Denmark). The Hgf^-/+^ Cdk4^R24C/R24C^ mouse strain was first generated as described previously [49]. Heterozygous hepatocyte growth factor (*Hgf)* overexpressing mice carrying a homozygous *Cdk4* (R24C) mutation (Hgf^-/+^Cdk4^R24C/R24C^ mice) as well as mice with wild type *Hgf* allele and the *Cdk4* (R24C) mutation (Hgf^-/-^ Cdk4^R24C/R24C^ mice) were kindly provided by Professor Tüting (University Hospital Magdeburg, Magdeburg, Germany). We performed further breeding in-house by crossing female Hgf^-/-^Cdk4^R24C/R24C^ and male Hgf^+/-^Cdk4^R24C/R24C^ mice. Approximately one‐third of mice in each litter were Hgf^+/-^Cdk4^R24C/R24C^ and the remaining were Hgf^-/-^Cdk4^R24C/R24C^. In experiments we used Hgf^-/-^Cdk4^R24C/R24C^, herein referred to as Cdk4^R24C^ mice, or Hgf^+/-^Cdk4^R24C/R24C^ mice. Hgf^+/-^Cdk4^R24C/R24C^ mice were distinguished by their black skin and coat due to overexpression of Hgf, herein referred to as Hgf-Cdk4^R24C^ mice. All animal experiments were performed upon approval by the regional Ethics Committees in Uppsala, Sweden (ethical permit C42/14 and 5.8.18-02686/2019). Housing conditions and experimental handling were performed according to the guidelines for animal experimentation and welfare at Uppsala University.

### Carcinogen induction

Mice were housed in groups of five per cage and N-Butyl-N-(4-hydroxybutyl) nitrosamine (OH-BBN) (Sigma-Aldrich cat. B8061) was added into drinking water at the final concentration of 0.05%. The drinking solution was freshly made and replaced twice weekly for 10 weeks. During this period the animals were weighed and examined twice weekly. The monitoring was performed every 24 hours after establishment of advanced palpable tumors and hematuria development. The humane endpoint was determined as 10 days of visible light hematuria or 5 days of complete hematuria or 15% body weight loss. The two experimental endpoints that were used to sacrifice the mice and perform several studies on the bladder tissue were 1) 10 weeks of carcinogens exposure and 2) palpable bladder tumor and humane (MIBC) endpoint.

### Bladder measurement and histopathology analysis

At 10-weeks carcinogen endpoint OH-BBN (female n = 5, male n = 6) and at MIBC endpoint (females n = 6, males n = 3) mice were sacrificed, bladders were harvested and emptied of urine, measured with a caliper and the ellipsoid formula “(4/3) x π x r1 x r2 x r3” was used to determine their volumes. Bladders as well as lungs and kidneys were fixed in Formaldehyde 4% buffered (Q Path® cat. 11699455) and embedded in paraffin. Sections of 4 μm thick were cut and stained with HE. The sections were examined by a pathologist for determining the presence and characteristics of malignant tissue, using healthy mouse tissue as reference in a genotype-, age- and sex-matched manner. The same criteria as for staging human bladder cancer were used. Digital microscopy images were acquired with a 3.3 M pixel CMOS camera (Olympus SC30, Olympus corp. Tokyo, Japan) and the image acquisition software CellSense v1.18 at 10x (200 μm scale bar), 20x (100 μm scale bar) and 40x (50 μm scale bar) magnifications.

### Generation of murine Hgf-Cdk4^R24C^ urothelial cancer cell lines

After 10 weeks of OH-BBN exposure mice were maintained while given only normal drinking water for one (n = 2 mice), five (n = 2 mice) or 10 (n = 1 mouse) weeks. At the designated time points mice were sacrificed, bladders were resected and digested under sterile conditions with Liberase TL (Roche, 2 ml PS/2.5 mg cat. 291959) for 15 min. Single cell suspensions were acquired via filtering in 70-μm cell strainers (Sigma cat. 431751) and seeded in T75 tissue culture treated flasks (Sarstedt) in RPMI 1640 medium supplemented with Glutamax (Thermo Fisher cat. 61870–010), 10% FBS (Gibco cat.11550356), Penicillin/ Streptomycin (Thermo Fisher cat. 15140–122), and cultured at 37°C, 5% CO2. Adherent cell fractions were separated from floating cells and cell cultures maintained for additional 16 weeks. Single cell colonies were isolated by limiting dilution in 96 well plates, expanded and mycoplasma tested. For determination of the doubling time cell lines were seeded in triplicates and cell counts acquired with an automated cell counter (Bio-Rad TC20) during the first four days were applied to the function y = a2Tx-b (a, b = parameters, T = doubling time). Additionally, CDK4 (R24C) mutation was confirmed by restriction enzyme digestion as previously described [26] with HindIII-HF endonuclease (New England Biolabs, US-MA, cat. R3104S).

### qRT-PCR of cell lines and urothelium

The expression of selected genes was analyzed in six new Hgf-Cdk4^R24C^ cell lines, MB49 cells and urothelium scrape from healthy Hgf-Cdk4^R24C^ bladder. Briefly, RNA was isolated using the Total RNA Purification Kit (Norgen Biotek cat. 17200) and DNase column treatment (Norgen Biotek cat. 25710) was performed according to the manufacturer’s instructions. The RNA concentration was quantified using NanoDrop Spectrophotometer (Thermo Fisher Scientific) and 1.5 μg of RNA was reverse-transcribed into cDNA in a 30 μl reaction using the iScript RT kit (Bio-Rad cat. 170–8890) according to the manufacturer’s instructions. Gene expression was determined using qRT-PCR with Sybr Green detection and PowerUp Master mix (Thermo Fisher cat. A25742) on a CFX384 Touch detection system (Bio-Rad). The primer pairs (S1 Table) were designed by us and ordered from Integrated DNA Technologies. The qPCR conditions consisted of 2 minutes initial activation at 50°C, 2 minutes denaturation at 95°C, followed by 40 cycles of 20 seconds denaturation at 95°C, 20 sec annealing at 55°C and 60 sec of extension at 72°C. After the completion of all cycles a final extension step for 10 minutes at 72°C was applied and melting curves were generated.

### Flow cytometry analysis of cell lines

Cells were harvested, washed in buffer (PBS containing 1.5% BSA) and antibody dilution 1:100 in 50 ul buffer were added. Incubation of 30 minutes in 4°C in the dark, was followed by 3 washes and 30 000 events were acquired in a CytoFLEX flow cytometer (Beckman Coulter) with a three-laser configuration (408, 488 and 640 nm). For the analysis of IFNγ induced stimulation, the 6 new cell lines as well as MB49 cells were cultured with or without IFNγ (10ng/ml) for 24h and expression of selected immune recognition cell surface markers (S2 Table) was analyzed by flow cytometry.

### The orthotopic MB49 model

The tumorigenic cell line MB49 (Mouse Bladder-49) was kindly provided by Dr K. Esuvaranathan, National University of Singapore, Singapore. The original stock vial was expanded in culture to a low passage clone and cryopreserved in liquid nitrogen as a primary cell line stock for rederivation purposes. Fig 2B, 2C and 2E shows the expression pattern of known cell adherence, urothelial and immune associated markers on the MB49 cell line. Cells used in the experiments were thawed and cultured for not more than 7 passages. The cell culture and the orthotopic MB49 model instillation were performed as previously described [54], the animals were sacrificed and tumors were harvested after 5 days. The anesthesia used was 75 mg/kg ketamine and 1 mg/kg medetomidine administered intraperitoneally and reversal was induced by 1 mg/kg atipamezole administered subcutaneously, both at 0.1 ml per 10 g of body weight. During the duration of the experiment the animals were weighed and examined twice weekly. Urine and serum were collected 1 day prior to and 5 days post intravesical instillation of MB49 cells. The humane endpoint for the orthotopic MB49 model was the same as for carcinogen induced bladder cancer: 10 days of visible low hematuria or 5 days of complete hematuria or 15% body weight loss. In the experiments performed no MB49 tumor bearing animals reached the humane endpoint.

### Single cell RNA sequencing (single-cell RNAseq) of whole bladders

Single cell transcriptomic analysis was performed to study the transcriptomic heterogeneity of tumor cells. Single cell analysis was performed for whole mouse bladders after a) 10 weeks of OH-BBN exposure and after b) progression to MIBC, as well as of c) healthy Hgf-Cdk4^R24C^ bladders, d) orthotopic MB49 tumor bearing (C57BL/6) bladders and e) healthy C57BL/6 bladders. On the 10-week endpoint Hgf-Cdk4^R24C^ tumors required pooling of samples due to low cell numbers per bladder (one pool of n = 4 females and one of n = 5 males). For the MIBC endpoint, one whole tumor bearing bladder per male and female were analyzed. Healthy Hgf-Cdk4^R24C^ bladders were pooled together (n = 4 males and n = 4 females). A pool of day 5 orthotopic MB49 tumors (pool of n = 5) and a pool of 5 healthy C57BL/6 bladders as control were also analyzed. Bladders were dissociated with the mouse tumor dissociation kit (Miltenyi cat. 130-096-730) according to the manufacturer’s instructions to create single cell suspensions. Samples were submitted to downstream procedures if viability was 75% or higher. Samples were processed using Chromium Single Cell 3’ GEM, Library & Gel Bead Kit v3 (10x Genomics cat. 1000092) according to the manufacturer’s instructions and sequencing performed with a NovaSeq6000 sequencer. Appropriate QC and integrity analysis were performed. Read alignment, barcode matrices and cell clustering were performed with the Cell Ranger 3.0 software. Gene expression analysis and visualization of the data was performed in R studio [55] using the Seurat 3.0 package [56] for R. One large urinary epithelium cell cluster (14386 cells) was identified in the dataset on the basis of single cell clustering at low resolution and use of the cell identity prediction tool of the PanglaoDB database [57] using differential gene expression. Subsequently, the urinary epithelium cells were extracted from the dataset as a subset and clustered anew. Then, cell clusters found exclusively in tumor bearing samples were extracted as subsets and clustering was performed in order to investigate whether heterogeneous tumor cells could be identified. The expression of Mki67 was used as a cancer cell proliferation marker gene and its expression on the chosen cell clusters was assessed to determine highly proliferating cells. The expression of *Ceacam1* was used as a marker of cancer cell stemness. MB49 cells were identified by expression of *Mki67*, *Vimentin* and Y-linked genes as these cells are originally derived from a male mouse and are implanted in female C57BL/6 bladders. The expression levels of a set of molecular subtype relevant genes were used to determine the molecular subtype profile tendency of each urinary epithelium cell cluster.

### Microhematuria detection and Proximity Extension Assay (PEA)

Collection of all urine samples was performed at the same time (between 9-11AM) to facilitate alignment of the data. Microhematuria was assessed at timepoints day 24, day 48 and day 70 of carcinogen exposure when visible hematuria was absent by using a semi-quantitative test (Combur Test UX, Roche) and the remaining urine was cryopreserved along with urine from the MIBC endpoint at -80°C for PEA analysis. Blood was collected at the 10-week endpoint and at the MIBC endpoint via tail vein incision, centrifuged at 10000 x g for 5 minutes, serum was collected and cryopreserved at -80°C. Urine and serum samples were randomized on 96-well plates and blinded PEA analysis was performed (Mouse Exploratory panel, Olink Proteomics, Uppsala, Sweden). Healthy control samples from age-, genotype-, sex-matched animals were used as reference for NPX values (Normalized Protein eXpression, Olink’s arbitrary unit). In the text, protein names are denoted in non-italic with a first capital letter and the rest lower cases for murine proteins, while human proteins are denoted in non-italic capital letters.

### Anti-PD1 treatment in animals with autochthonous bladder tumors

To assess the therapeutic effect of anti-PD1 therapy on OH-BBN exposed Hgf-Cdk4^R24C^ male and female animals, one week after the 10-week exposure period, animals received six intraperitoneal administrations of 100 μg InVivoMAb anti-mouse PD-1 (CD279), Clone RMP1-14 (BioXCell cat. BE0146) in 50 μl PBS every 4.5 days for 3 weeks. Animals were monitored twice weekly or every 24h when signs of sickness appeared. Mice were sacrificed at humane endpoints (maximum 10 days of low hematuria or 5 days of complete hematuria or 15% body weight loss). Six out of 10 animals (3 out of 4 males, 3 out of 6 females) that received anti-PD1 therapy suffered sudden death prior reaching the humane endpoint. The survival of the treated animals was compared to historically recorded OH-BBN exposed, but untreated (6 female and 7 male) control Hgf-Cdk4^R24C^ mice which were also monitored daily upon appearance of palpable tumors and sacrificed at the humane endpoint.

### Tumorigenicity of new tumor-derived cell lines and *in vivo* anti–PD-1 or CpG ODN 1668 treatment of subcutaneous tumors

The tumorigenicity of the 6 new Hgf-Cdk4^R24C^ urothelial cancer cell lines was assessed in 2 or 4 mice by subcutaneous inoculation of 5x10^6^ cells to C57BL/6 female mice or 2x10^6^ cells to Hgf-Cdk4^R24C^ female mice. Animal weight and tumor growth were assessed twice a week by caliper measurement for two months (experimental endpoint set to assess tumorigenicity of cell lines). To assess the therapeutic potential of anti-PD-1 or CpG ODN 1668, the only tumorigenic cell line, 74/7, was inoculated to the flanks of Hgf-Cdk4^R24C^ mice (8 females and 7 males per group, 3–5 months old). On day 0 the mice received subcutaneous inoculum of 2x10^6^ cells. Animals were monitored, weighed and tumors were measured with a caliper twice a week and the ellipsoid formula “(4/3) x π x r1 x r2 x r3” was used to determine their volumes. From day 10 the mice were treated with 5 doses of 50 μg anti PD-1 (BioXCell cat. BE0146) peritumorally every third day or with 6 doses of 50 μg CpG ODN 1668 (tlrl-1668-5, Invivogen) peritumorally every third day. The control group received an equal number of PBS injections. For survival analysis, the animals were monitored until the tumor size reached the volume of 1 cm^3^ which was the determined humane endpoint measure for subcutaneous tumors.

### Statistical analysis and illustrations

Statistical significance of survival curves was determined by Log-rank (Mantel-Cox) testing. For direct comparisons of groups non-normal distribution of the data was assumed (due to small numbers of groups) and therefore non-parametric comparisons were performed (Mann-Whitney). Error bars represent standard error of the mean. Power analysis to determine the number of animals required for experimental assessment of anti-tumor therapeutic effect (increased survival/reduced tumor size) (alpha 0.05, power 80%), indicated that the sample size should be 5 mice per group. Hence, the study was designed to be sufficiently powered for the primary endpoint of determining if there is a difference among groups in anti-tumor response. In occasions where transgenic mice were used this number was sometimes lower due to the low ratio (below Mendelian ratio) of birth of Hgf-Cdk4^R24C^ mice. For statistical analysis of proteomic data in the form of NPX values, Mann-Whitney tests were used for comparisons between groups. For analysis of longitudinal proteomic alterations across time the Generalized Least Square model was applied. Volcano plots were generated with R studio [55]. KEGG pathway analysis was performed with the function ‘Proteins with Values/Ranks’ in the online database STRING [58]. Graphical workflow illustrations were created with BioRender.com. Graphs were generated with GraphPad Prism 7.0 and R studio 1.1.463 [55].

## Results

### Establishment of OH-BBN induced urothelial tumorigenesis in Hgf-Cdk4^R24C^ mice

**Fig 1:**
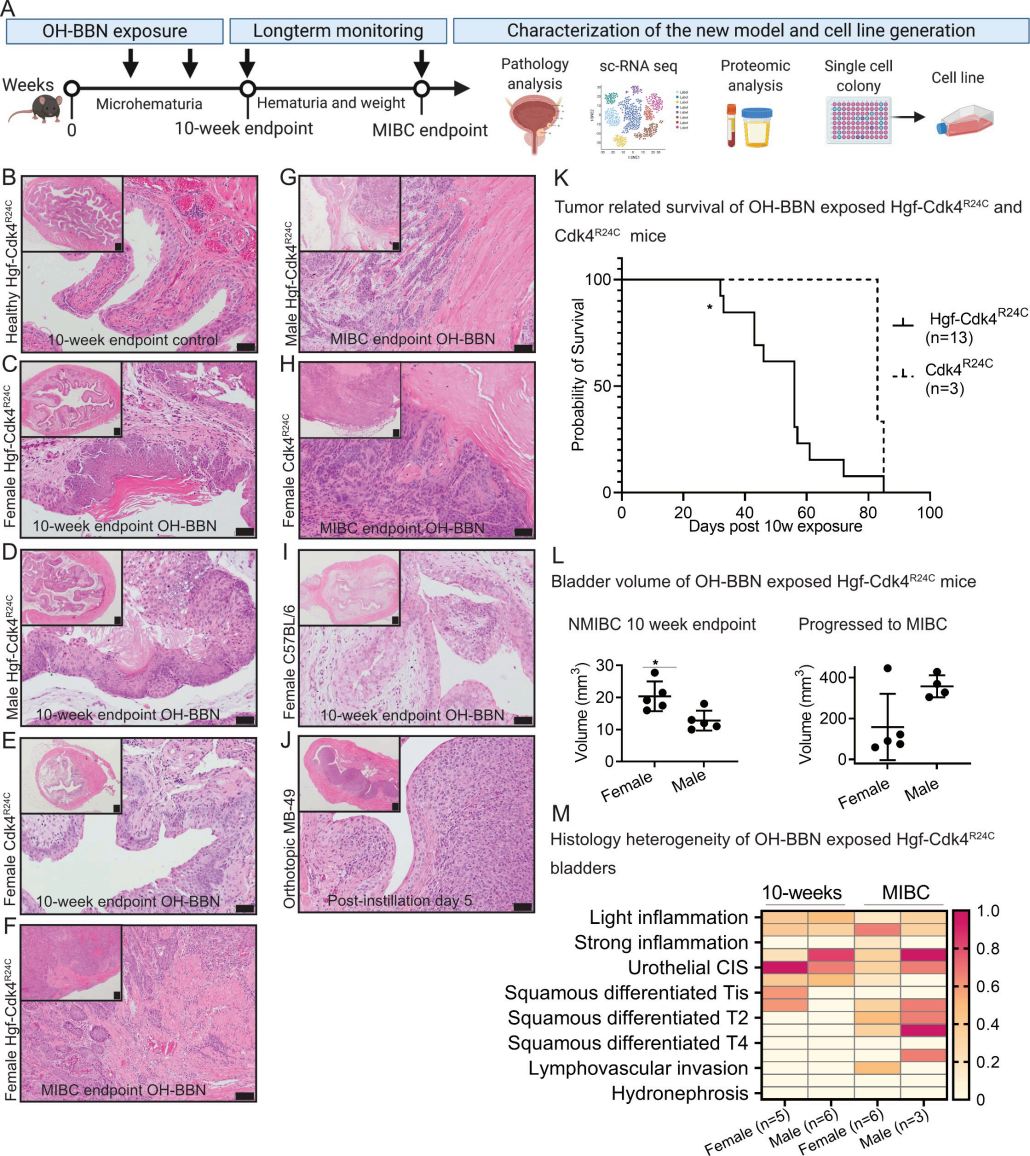
Development and characterization of the new Hgf-Cdk4^R24C^ urothelial cancer model. (A) Workflow summary of the model induction, timepoints setup and subsequent analyses. Representative images of bladder sections from (B) healthy female Hgf-Cdk4^R24C^, the 10-week OH-BBN induced (C) female Hgf-Cdk4^R24C^, (D) male Hgf-Cdk4^R24C^ and (E) female Cdk4^R24C^ mice. Representative images of the MIBC endpoint bladder sections from (F) female Hgf-Cdk4^R24C^, (G) male Hgf-Cdk4^R24C^, (H) female Cdk4^R24C^ mice, and of (I) 10-week OH-BBN induced female C57BL/6 mice as well as (J) the orthotopic MB49 model at day 5. (Scale bars indicate 200 μm in all tissue overview images, 50 μm in enlarged images B-E and I-J, and 100 μm in enlarged images F-H.) (K) Survival was significantly shorter for Hgf-Cdk4^R24C^ than for Cdk4^R24C^ mice following 10-weeks OH-BBN exposure until reaching the humane endpoint due to tumor burden (p = 0.0377). (L) Bladder volume comparison between female and male Hgf-Cdk4^R24C^ mice at the 10-week (p = 0.0317) and the MIBC endpoints (p = 0.1905). (M) A summary of heterogeneous histopathology findings on each murine bladder and kidney at the two endpoints (10-weeks OH-BBN female n = 5, male n = 6, MIBC females n = 6, males n = 3) in Hgf-Cdk4^R24C^ mice. The heatmap scale indicates the fraction animals in each group that presented each histology.

Administration of the pro-carcinogen OH-BBN (metabolized to active carcinogen in the liver and in the bladder) is used to selectively induce urothelial cancer in animal models [17]. Herein, we used tumor induction by 10 weeks of exposure to 0.05% OH-BBN in drinking water in Hgf-Cdk4^R24C^ or Cdk4^R24C^ mice. For some parameters we also compared the tumorigenesis in C57BL/6 female mice using either OH-BBN induced tumors or orthotopically implanted syngeneic MB49 tumors. In a pilot experiment, we compared tumor induction in Hgf-Cdk4^R24C^ weaned (4–6 weeks old; before full immune system development) and adult (10–14 weeks old, with fully developed immune system) female mice. Histopathology examination of bladders showed that carcinogen exposure of older mice was associated with increased immune cell infiltration, in contrast to mice exposed at weaning age that presented with low or no inflammation (S1A Fig). Since inflammation is an important disease component in humans, we used adult mice for further studies. Bladders were collected for histology analyses at two timepoints: 1) 10 weeks of carcinogen exposure and 2) at development of symptoms of advanced urinary bladder tumor (humane endpoint due to tumor burden) (Fig 1A). Pathology assessment revealed that non-carcinogen exposed female Hgf-Cdk4^R24C^ mouse bladder histology was normal (Fig 1B), whereas carcinogen treated Hgf-Cdk4^R24C^ females displayed a urothelial cancer of Tis-T1 stage with squamous differentiation (Fig 1C) which was more advanced than for transgenic Hgf-Cdk4^R24C^ males (Fig 1D) or Cdk4^R24C^ littermates (Fig 1E).

Next, progression and survival of the tumor-bearing Hgf-Cdk4^R24C^ and Cdk4^R24C^ mice were investigated under active surveillance until reaching the tumor burden related humane endpoint. Histopathology analysis upon active surveillance revealed that in transgenic mice, tumors progressed to MIBC with squamous differentiation (Fig 1F and 1G). For wildtype mice, MIBC rarely develops after 10 weeks of OH-BBN exposure [17], thus C57BL/6 mice were not investigated towards progression to MIBC. However, for the first endpoint of 10 weeks exposure as a comparison we found that female C57BL/6 mice developed urothelial dysplasia with or without focal carcinoma in situ (CIS) (Fig 1I), which was not as advanced as in transgenic female or male mice (Fig 1C and 1D). In contrast, orthotopic MB49 tumors presented with more consistent and homogenous histology than OH-BBN induced tumors, with low degree of inflammation (Fig 1J). The transgenic mice reached a humane endpoint within 5–11 weeks of active surveillance after ending carcinogen exposure, while the survival difference among sexes did not reach statistical significance (Median survival: Female: 56 days; Male: 43 days). The survival of Cdk4^R24C^ mice upon progression to MIBC was significantly longer (p = 0.0377) compared to their Hgf-Cdk4^R24C^ littermates (Fig 1K) (Median survival: Hgf-Cdk4^R24C^: 56 days; Cdk4^R24C^: 83 days). Based on this we pursued our studies with the Hgf-Cdk4^R24C^ mice which seem to progress faster than mice carrying the Cdk4^R24C^ mutation alone without overexpression of Hgf. Interestingly, after only 10 weeks of carcinogen exposure, female Hgf-Cdk4^R24C^ bladders presented with a noted enlarged volume than males, whereas at the MIBC timepoint male bladders tended to be larger than for females without reaching statistical significance (Fig 1L). The summary of detailed histopathology analysis for Hgf-Cdk4^R24C^ mice is presented in Fig 1M. In half of the female Hgf-Cdk4^R24C^ bladders with MIBC, lymphovascular invasion by tumor cells was observed, but none of the mice developed distant metastasis in lungs or kidney tumors. Despite the advanced tumor stage noted in a few mice (T4), no hydronephrosis was observed.

### Development of Hgf-Cdk4^R24C^ urothelial cancer cell lines

**Fig 2:**
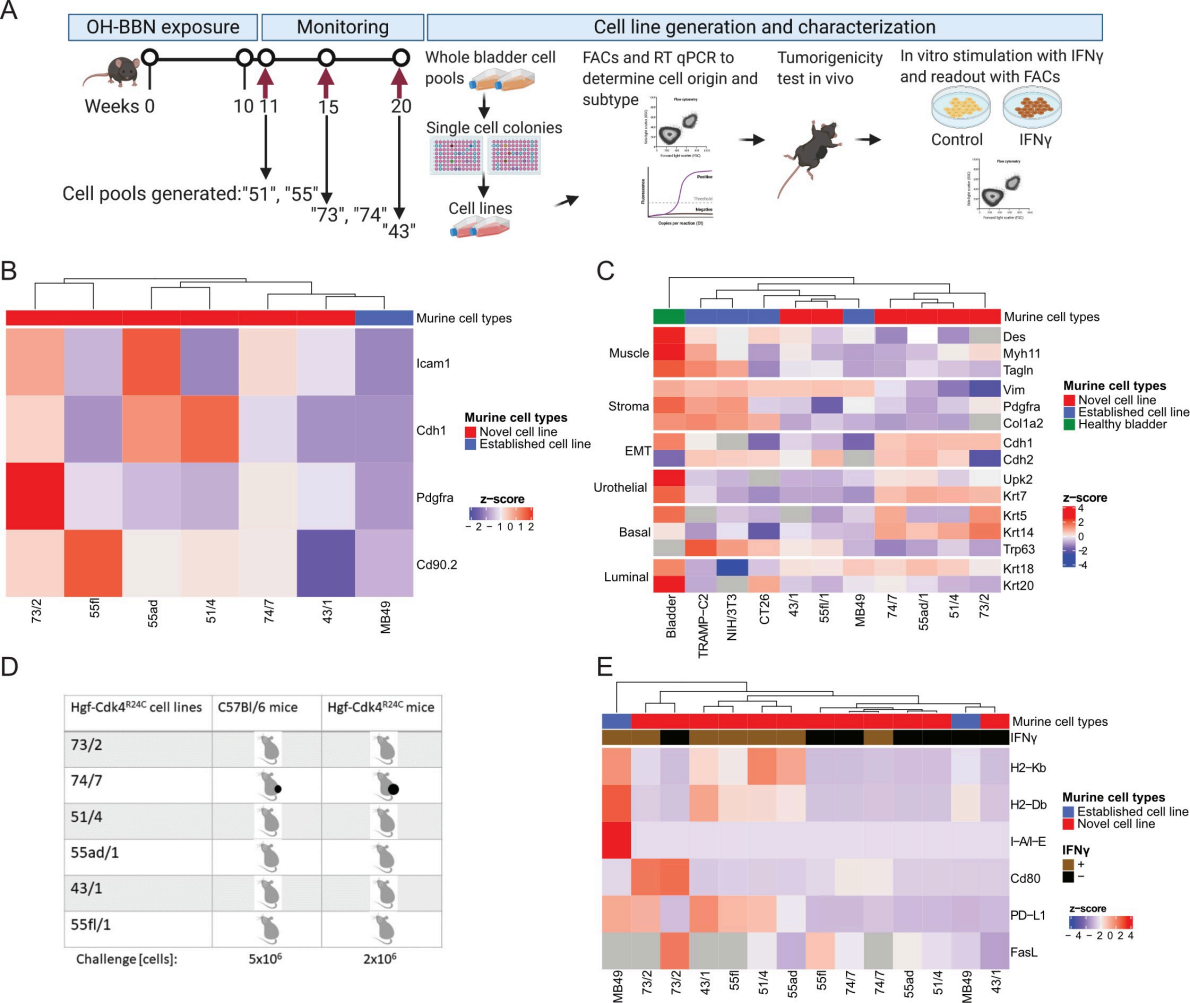
Development and characterization of the cell cultures developed from bladder tumors in Hgf-Cdk4^R24C^ mice. (A) For cell line generation, female Hgf-Cdk4^R24C^ mice were exposed for 10–11 weeks to OH-BBN in drinking water. After OH-BBN discontinuation, mice were monitored for 1, 5 or 10 weeks and sacrificed. Six single cell colonies were randomly selected and characterized by (B) expression of surface markers by flow cytometry or (C) qRT-PCR to determine the cell origin and molecular profile of the cells in comparison to established cell lines or healthy bladder urothelium. (D) Only the 74/7 cell line formed tumors after subcutaneous inoculation to C57BL/6 or Hgf-Cdk4^R24C^ mice in doses of 5 or 2x10^6^ cells, respectively. The new cell lines and MB49 cells were cultured with or without IFNγ (10 ng/ml) for 24h and (E) the expression of cell surface immune recognition markers was analyzed by flow cytometry (normalized to unstained control cells).

To develop a novel *in vitro* model of bladder cancer, we generated cell lines originating from Hgf-Cdk4^R24C^ urothelial tumors using whole bladder cell pools (Fig 2A). Thereafter, to exclude the potential presence of cells of non-malignant origin in the cell cultures, we analyzed the expression of epithelial–mesenchymal transition (Icam1 and E-cadherin) and fibroblast (Pdgfra and Cd90.2) markers by flow cytometry (Fig 2B). To further determine the cell origin and the molecular profile of the new cell lines, we examined the expression of genes that characterize muscle (*Des*, *Myh11*, *Tagln*), stroma (*Vim*, *Pdgfra*, *Col1a2*), epithelial mesenchymal transition (EMT) (*Cdh1*, *Cdh2*), urothelial (*Krt7*), basal (*Krt5*, *Krt14*, *Trp63*) and luminal (*Krt18*, *Krt20*) cells and compared the novel cell lines with established cell lines and healthy Hgf-Cdk4^R24C^ urothelium (Fig 2C). All of the novel cell lines displayed a lack of or very low expression of genes related to muscle and stroma when compared to healthy bladder, the urothelial cell line MB49 or the non-urothelial cell lines TRAMP-C2, NIH/3T3 and CT26. All cell lines showed that they are undergoing or have completed EMT through expression of lower levels of *Cdh1* and higher levels of *Cdh2* compared to healthy bladder tissue, with the exception of 73/2 cell line. Expression of two general urothelial markers (*Upk2*, *Krt7*) was stronger in healthy bladder followed by the new cell lines 74/7, 55ad/1, 51/4 and 73/2, while a tendency towards basal molecular profile was displayed by these cell lines as assessed by expression of *Krt5* or *Krt14*. *Trp63* was strongly expressed by non-urothelial cell lines followed by the new 43/1 and 55fl/1 cell line. All new cell lines and MB49 cells expressed moderate or lower levels of the luminal gene markers *Krt18* and *Krt20* in comparison to healthy bladder. Hierarchical clustering based on the expression of these genes revealed that the novel cell lines and MB49 cells were more similar to each other than to healthy bladder (Fig 2C). The novel cell lines expressed the *Cdk4 (R24C)* transgene (S3A Fig), and their doubling time was determined to be between 14-31h (S3B Fig). Next, *in vivo* growth of all new cell lines by subcutaneous engraftment in wild type and transgenic mice was assessed. Of these, only the 74/7 cell line developed tumors *in vivo* in Hgf-Cdk4^R24C^ and C57Bl/6 mice (Fig 2D). To explain the lack of tumorigenicity in immunocompetent mice of all novel cell lines except for 74/7, the expression of immune recognition receptors was examined before and after IFNγ stimulation *in vitro* using flow cytometry (Fig 2E). As previously shown [59], MB49 cells that were used for comparison in this experiment were MHC class I positive, and upon IFNγ stimulation upregulated MHC class I, II and PD-L1 (Fig 2E). Without IFNγ stimulation the five non-tumorigenic new cell lines were MHC class I and II deficient, PD-L1^low/neg^, CD80 deficient (except for 73/2) and FasL^low^. However, they displayed increased MHC class I and/or PD-L1 expression after exposure to IFNγ for 24h *in vitro* (Fig 2E). Conversely, the 74/7 tumorigenic cell line showed no response to *in vitro* IFNγ stimulation (Fig 2E) providing evidence that the other cell lines did not form tumors *in vivo* due to immune rejection.

### Single cell transcriptomic analysis identified distinct transformed tumor cell clusters

**Fig 3:**
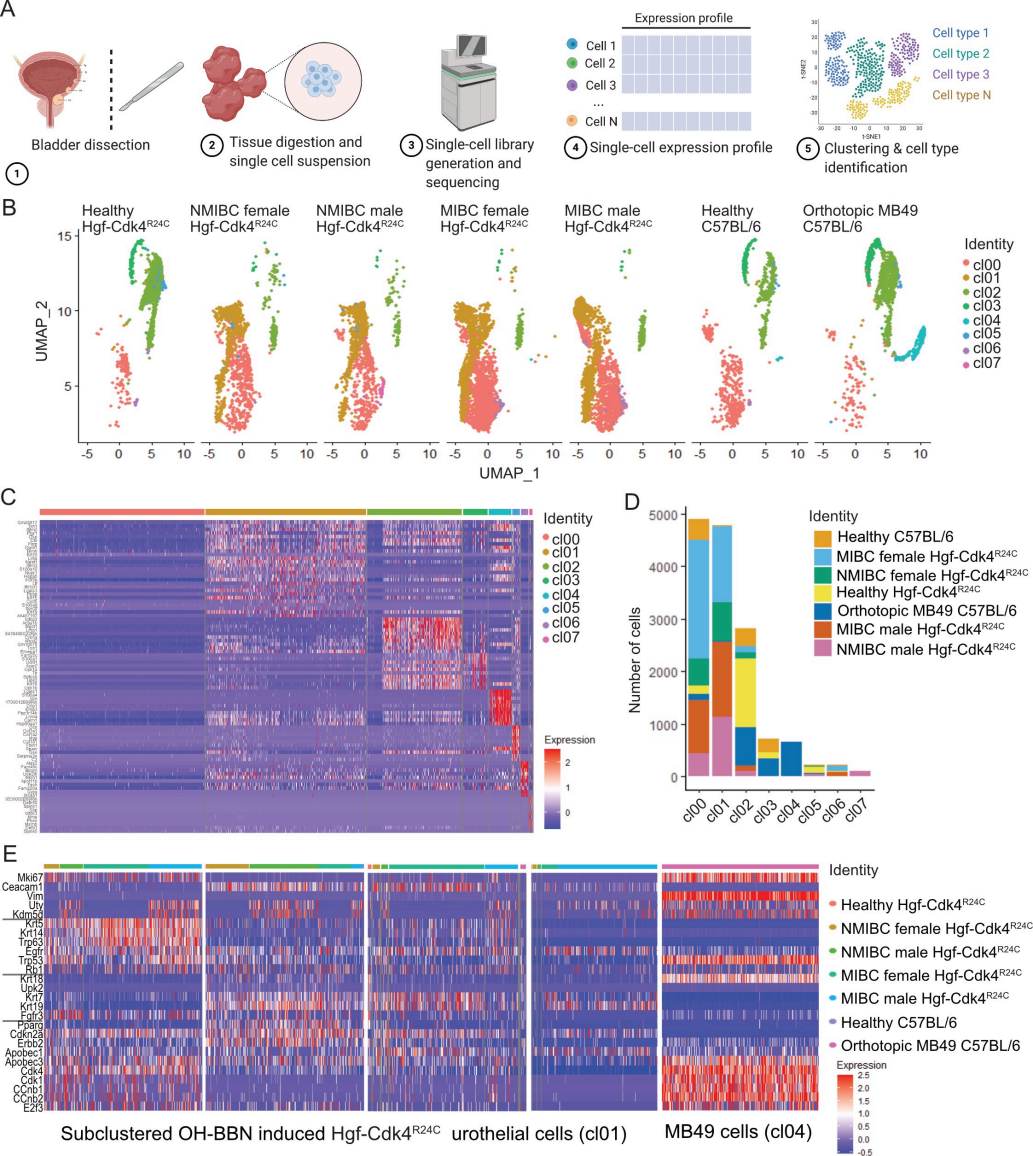
Single cell transcriptomic analysis of the tumor bearing and healthy bladders. (A) Workflow of the single-cell RNAseq, and (B) UMAP projection of all urinary epithelium cell clusters (cl00-cl07) in all of the samples analyzed by single-cell RNAseq. (C) Transcriptomic heterogeneity of the identified urothelial cell clusters and (D) number of cells from each cluster in each bladder analyzed for Hgf-Cdk4^R24C^ or C57BL/6 mice. Out of all cell clusters in Hgf-Cdk4^R24C^ and MB49 tumor bearing bladders (B, C, D), cl01 which is present only in OH-BBN induced Hgf-Cdk4^R24C^ bladders and cl04 which is present only in MB49 tumor bearing bladders, were further subsetted/subclustered and the expression of (D) tumor or cancer stem cell marker genes, as well as molecular subtype relevant genes is visualized for each model.

To our knowledge there is very limited number of studies that have characterized healthy epithelial cells of the human and mouse bladders [60], as well as tumor, stroma and immune cells from human bladder tumors [61] using single cell transcriptomics. Molecular subtyping for human bladder cancer is performed by bulk DNA or RNA sequencing of tumor biopsies. To better visualize the intratumoral transcriptomic heterogeneity in bladder cancer, we performed single-cell RNAseq analysis on murine OH-BBN induced Hgf-Cdk4^R24C^ urothelial tumors as well as on orthotopic MB49 tumors (Fig 3A). The urinary epithelium cells were extracted from the dataset and analyzed as described in Materials and Methods. Initially eight cell clusters of urinary epithelium origin with transcriptomic heterogeneity were derived, cl00-cl07 (Fig 3B and 3C, S1 File). Cluster cl01 was enriched only in OH-BBN induced Hgf-Cdk4^R24C^ bladders and cl04 only in MB49 tumor-bearing bladders (Fig 3D). Cells belonging to cl01 and cl04 were subclustered again and then characterized exclusively in terms of gene expression of proliferation markers, cancer cell stemness and molecular subtype profile markers. Upon re-clustering, four clusters emerged from cl01 (Fig 3E): 1) a highly proliferating cluster (based on *Mki67* expression) with basal/squamous and cell cycle deregulated profile gene expression, while some cells of this cluster in NMIBC also displayed *Fgfr3* expression, 2) a cancer stem cell cluster (based on *Ceacam1* expression) with mixed expression of subtype-related genes and higher *Pparg* expression than the other clusters, 3) a cancer stem cell cluster enriched in female MIBC that displayed a heterogeneous profile of subtype-related genes expression, and 4) a cluster with a heterogeneous *Ceacam1* expression that was present mainly in male MIBC including cells with high and low *Egfr* expression, but overall low expression of subtype-related genes. The MB49 cell cluster (Fig 3E, right) displayed a strong cell cycle dysregulation gene expression profile, similar but higher in the expression scale compared to the highly proliferating Hgf-Cdk4^R24C^ tumor cell cluster, and with visibly higher expression of *Tp53* compared to all other Hgf-Cdk4^R24C^ clusters. These findings suggest that urothelial cells that are present only in carcinogen induced tumors, but not in healthy bladders, may or may not present high proliferation or cancer stemness, while still contributing to the tumor burden and possibly to different subtype profiles.

### Distinct protein profiles of serum and urine among bladder cancer models

Histopathology analysis depicted strain specific differences in OH-BBN induced bladder cancer development in wild type transgenic mice, while single-cell RNAseq showed differences in molecular profile between the MB49 and the OH-BBN induced Hgf-Cdk4^R24C^ model. To further investigate differences of the systemic protein profile and local tumor microenvironment among the different models, we performed a targeted proteomic profiling on liquid biopsies.

#### Serum

**Fig 4:**
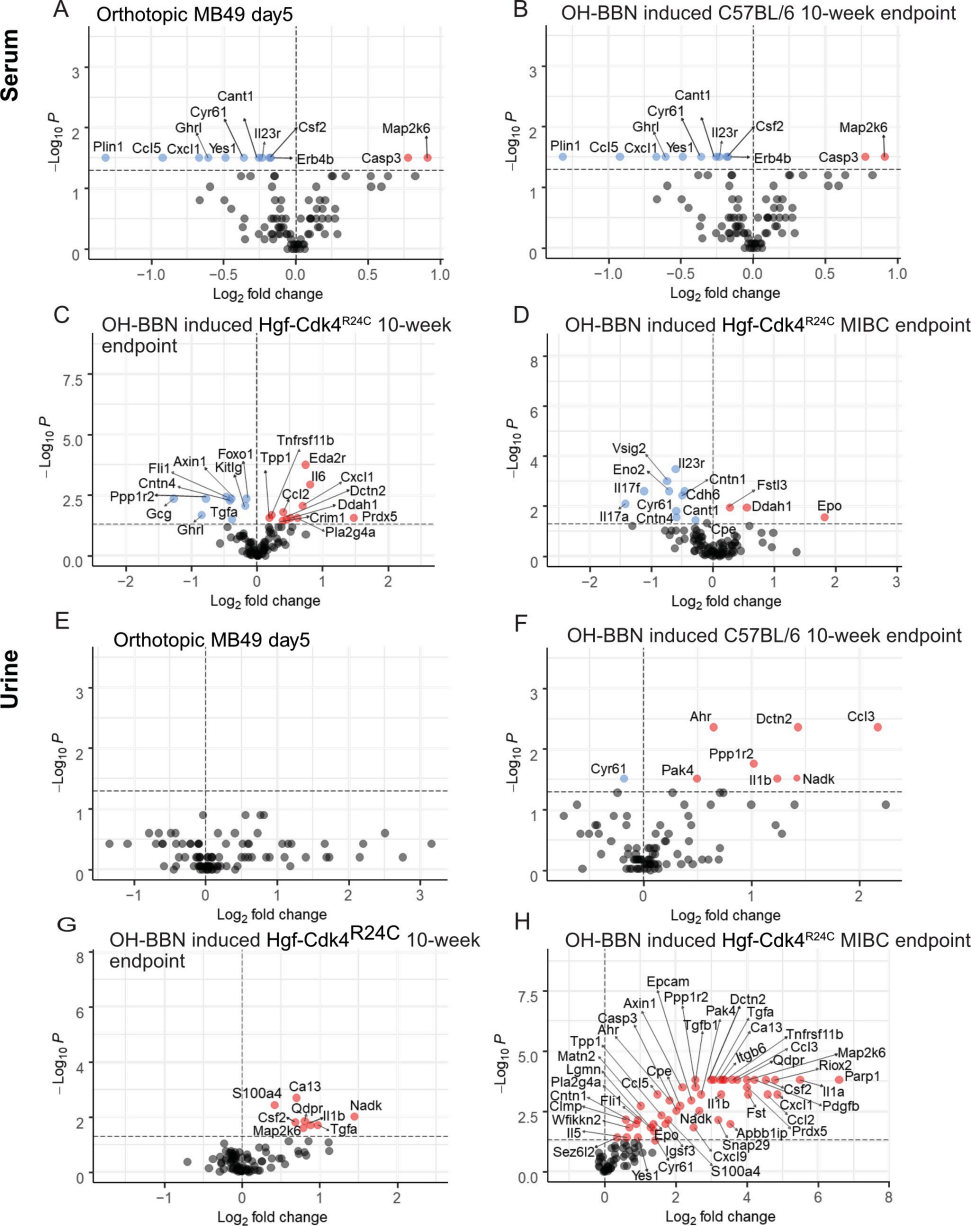
Protein signatures in serum and urine in three murine urothelial cancer models. Orthotopic MB49 model (day 5), carcinogen induced wild type female C57BL/6 (10-week endpoint) and carcinogen induced male and female Hgf-Cdk4^R24C^ (10-week and MIBC endpoint). Significantly altered serum proteins in (A) MB49 orthotopic model (n = 6), (B) Carcinogen induced female C57BL/6 mice (n = 6), (C) 10-week endpoint Hgf-Cdk4^R24C^ mice (n = 13), (D) MIBC endpoint Hgf-Cdk4^R24C^ mice (n = 8) compared to respective healthy controls (n = 6–10). Levels of significantly changed protein levels in the urine samples of (E) MB49 orthotopic model (n = 4), (F) Carcinogen induced female C57BL/6 mice (n = 6), (G) 10-week endpoint Hgf-Cdk4^R24C^ mice (n = 18), (H) MIBC endpoint Hgf-Cdk4^R24C^ mice (n = 8) compared to respective healthy controls (n = 4–13). Fold change for each protein was calculated as 2^(mean NPX cancer—mean NPX healthy control)^. Colored circles indicate significance level of p<0.05; red for increased proteins and blue for decreased proteins for cancer model compared to healthy controls, grey for proteins below the significance level.

When comparing serum signatures from the two OH-BBN induced strains (10-week endpoint) and the orthotopic MB49 model (day 5) (Fig 4) a mutual decrease of Ghrl was identified among the three models. Decreased levels of Plin1 were seen in wild-type OH-BBN exposed animals and MB49 day 5 orthotopic tumor bearing animals when compared to healthy controls of respective model (Fig 4A–4C). Moreover, the orthotopic MB49 model displayed decreased Ccl5, Cxcl9 and Cxcl1 levels, suggesting a less immunogenic systemic impact of the tumors. In this model, we further observed increased levels of the anti-proliferative cell cycle regulator Map2k6 and the apoptosis mediator Casp3 (Fig 4A). Additionally, an increased concentration of the apoptosis marker Parp1 was observed in wild type mice (Fig 4B) and increase of the tumor suppressor transcription factor Eda2r was seen in transgenic animals when compared to healthy controls of respective model (Fig 4C). Interestingly, Il6 levels were decreased in C57BL/6, but increased in the transgenic animals. This inverse correlation could suggest a polarized early systemic immune response during the time of tumor establishment in the different strains. In line with this observation, the pro-inflammatory cytokines Csf2, Il1β and Il5 were downregulated in the serum of C57BL/6 mice. At the MIBC endpoint serum of transgenic mice displayed increased Epo, nitric oxide and tumor growth regulator Ddah1, and Fslt3.

#### Urine

No significantly altered protein levels were detected in the inoculated MB49 mice at day 5 in urine when compared to urine before instillation (Fig 4E). However, the urine profile of OH-BBN induced transgenic and wild type mice were distinct at the 10-week endpoint (Fig 4F and 4G). Contrary to the serum profile (Fig 4B), urine from OH-BBN induced C57BL/6 mice displayed increased levels of the pro-inflammatory cytokines Ccl3 and Il1β, as well as an upregulation of tumor promoting Ppp1r2, Pak4, tumor growth marker Nadk, and Dctn2 (Fig 4F). Also, increased level of Ahr was detected in wild type OH-BBN induced mice. The transgenic mice displayed increased pro-inflammatory response markers linked to the fatty acid metabolism/prostaglandin pathway at 10-week endpoint, such as S100a4 and Il1β (Fig 4G). In MIBC the fatty acid/prostaglandin pathways remained as an upregulated signature along with other pro-tumorigenic factors (Fig 4H), such as Pdgfb and Tgfb1 as well as signals of an active DNA repair machinery, such as Parp1 and Apbb1ip.

### Proteomic analysis of urine and serum from early carcinogenesis to progression in male and female mice

**Fig 5:**
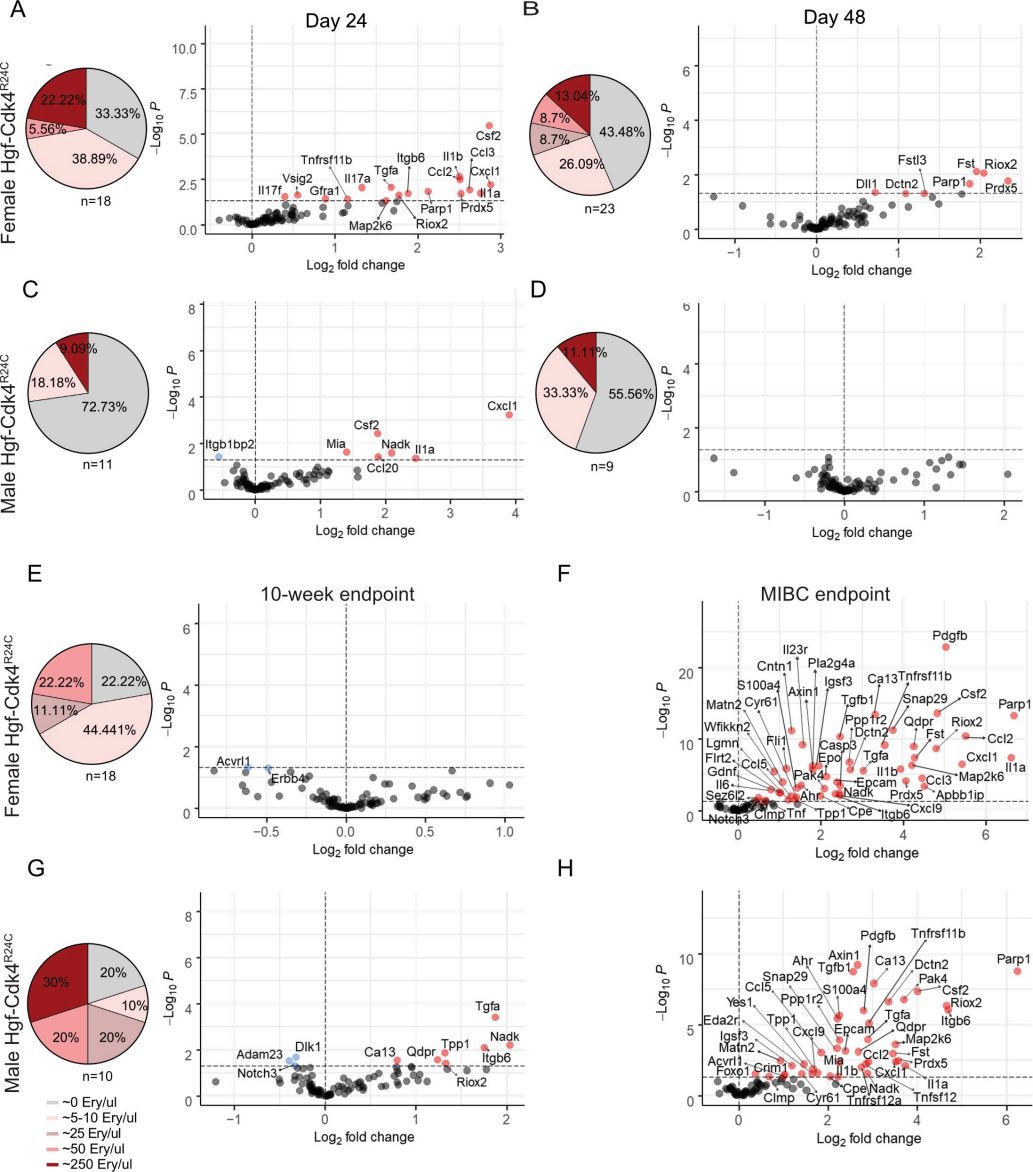
Microhematuria levels and sex specific protein alterations in urine over time in Hgf-Cdk4^R24C^ OH-BBN induced mice depict events of carcinogenesis, early immune response and tumor progression. From day 24 until the 10-week endpoint a percentage of animals presented none, low or high levels of microhematuria, expressed as erythrocytes/μl. At the MIBC endpoint gross hematuria was noted and therefore no microhematuria measurements were performed. Day 24 [(A) Females: n = 11 cancer, n = 3 healthy. (C) Males: n = 7 cancer, n = 3 healthy], day 48 [(B) Females: n = 11 cancer, n = 3 healthy. (D) Males: n = 6 cancer, n = 3 healthy], 10-week endpoint [(F) Females: n = 11 cancer, n = 6 healthy. (H) Males: n = 7 cancer, n = 7 healthy], MIBC endpoint [(G) Females: n = 5 cancer, n = 4 healthy. (I) Males: n = 3 cancer, n = 4 healthy]. Repeated measurements were analyzed by a generalized least square (GLS) to model the effect of gender on the disease at a specific time point. Fold change was calculated as 2^(mean NPX cancer—mean NPX healthy control)^. Colored circles indicate significance level of p<0.05; red for increased proteins and blue for decreased proteins for each time point compared to healthy controls, grey circles show proteins below the significance level.

An obstacle in studying human clinical material is performing repeated sample analysis due to logistic, ethical and feasibility constraints. Studies capturing early tumorigenesis are often too costly to perform as a large number of study subjects are required to capture tumor development. Bladder cancer patients at high risk of progression (based on histopathology and clinical parameters) undergo radical surgical interventions to prevent metastatic disease, limiting clinical sampling post that stage. However, an animal model of carcinogen induction can capture the timeline of early carcinogenesis as well as of the NMIBC and MIBC stages. A number of male and female OH-BBN induced Hgf-Cdk4^R24C^ animals displayed microhematuria already at day 24, but also throughout day 48 and the 10-week endpoint (Fig 5). The detection of microhematuria was used as a marker of early tumor lesion formation and urine collected at those time-points was used for proteomic analysis. Microhematuria or hematuria was not observed in day 0 samples and neither in health matched controls at any time-point. After the 10-week endpoint, animals displayed irregular hematuria until the progression to MIBC which resulted in weight loss, gross hematuria and palpable bladder tumors.

Fig 5A–5H outlines the proteomic profile during the early tumor development phase in male and female mice. Early protein alterations were present already at day 24 (3.5 weeks) of carcinogen exposure. The number of elevated proteins in urine at day 24 was larger in females (17 proteins) (Fig 4A) compared to males (6 proteins) (Fig 5C). Functional enrichment of the upregulated proteins in males and females using STRING [58] revealed pathways and activities associated with immune and stress responses, inflammation and cytokine regulation, leukocyte homing, apoptotic processes and cancer development (S2–S5 Files). Interestingly, most upregulated proteins at day 24 re-surfaced at the MIBC time-point whereas they were not detected at day 48 and 10-week endpoint (Fig 5B, 5D, 5E and 5G). An intersection analysis revealed several overlapping up-regulated proteins at early time points and the MIBC endpoint between sexes (S2E Fig), while hierarchical clustering based on significantly altered proteins reveals that male and female samples are partially clustered together by sex although the number of samples might be too low to allow this assumption (S2A–S2D Fig).

### Effects of anti-PD1 and CpG ODN immunotherapy in Hgf-Cdk4^R24C^ autochthonous and subcutaneous tumors

**Fig 6:**
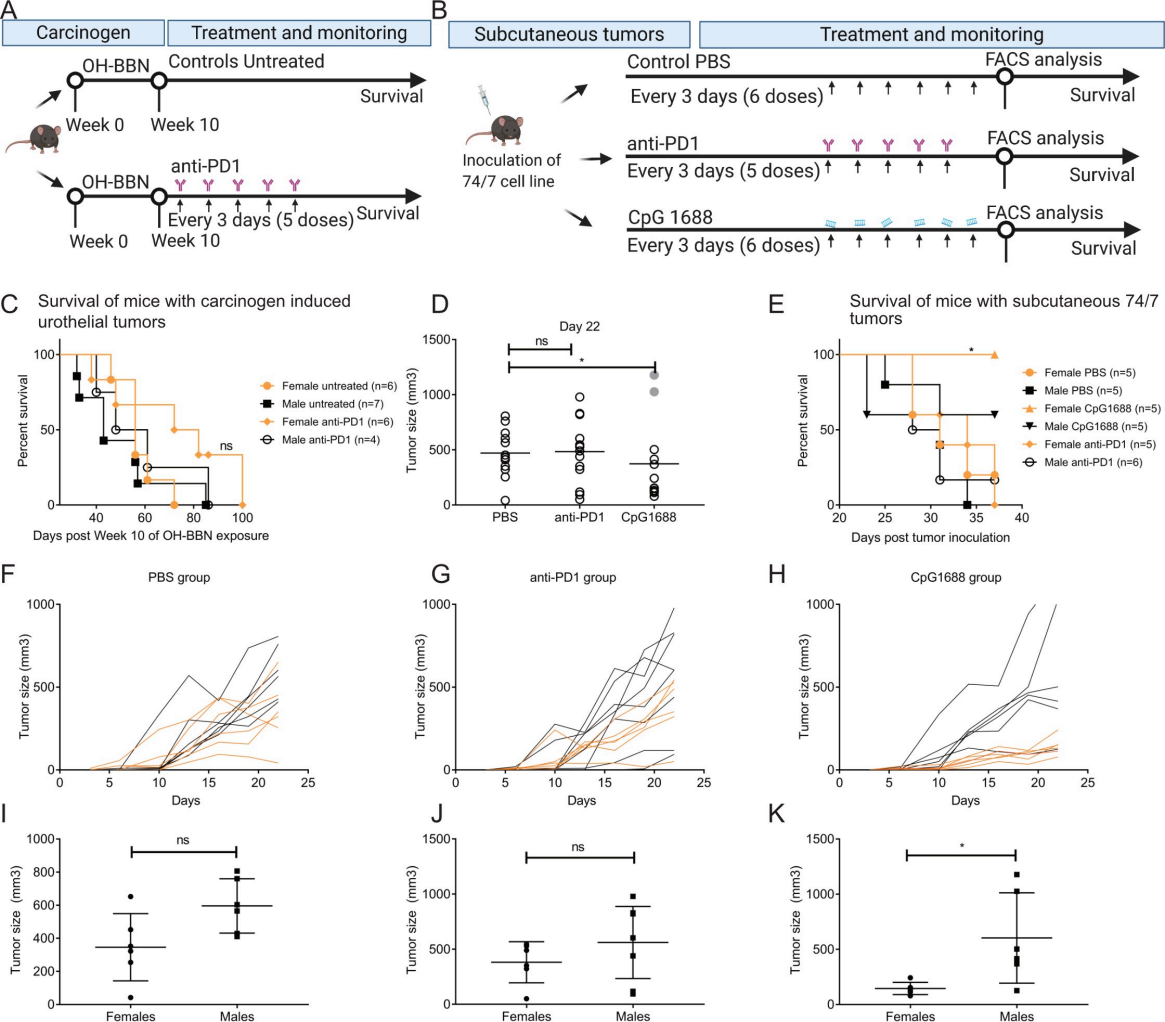
Experimental design and immunotherapy outcomes of OH-BBN induced or engrafted Hgf-Cdk4^R24C^ tumors. (A) In OH-BBN induced mice, the anti-PD1 therapy was administered intraperitoneally twice a week for 3 weeks (6 doses 100μg/mouse) with onset at one week after the disruption of carcinogen exposure, while untreated animals did not receive mock therapy. (B) 74/7 tumors were inoculated and treated with PBS, anti-PD1 (5 doses) or CpG 1668 (6 doses) administered peritumorally every third day starting at day 10 post inoculation. (C) Survival curves were not statistically different (p = 0.2406) in carcinogen induced animals upon anti-PD1 therapy. (D) The size of subcutaneous 74/7 tumors at day 22 was significantly reduced after CpG 1688 treatment (p = 0.0138), while (E) female mice treated with CpG 1688 survived significantly longer (p = 0.05) than the other groups. (F-H) Tumor size comparison between female (orange lines) and male (black lines) animals over time and (I-K) at day 22 showed that females receiving CpG 1688 therapy displayed significantly reduced tumor size by day 22 (p = 0.026).

To investigate whether or not anti-PD1 therapy can hinder progression to MIBC in Hgf-Cdk4^R24C^ mice with OH-BBN induced NMIBC we assessed it in this model (Fig 6A). No significant difference in survival was noted in response to anti-PD1 as compared to control survival measurements in the same strain (Median survival: Female untreated: 56 days; Male untreated: 43 days; Female anti-PD1: 77 days; Male anti-PD1: 54,5 days) (Fig 6C), although a trend of an increased median survival in both treated and untreated females was noted compared to male counterparts. Unexpectedly, many anti-PD1 treated animals died spontaneously before reaching the humane endpoint (included in the survival analysis shown), in contrast to the untreated group. Therefore, tumor measurement or other examinations of these animals were not performed. The repeated occurrence of spontaneous deaths could indicate other complications such as tumor spread, (uro)sepsis, anti-PD1 side effects, etc. Although there are no reports of direct immunotoxicity of anti-PD1 therapy in the Hgf-Cdk4^R24C^ mouse strain in previous studies [62], it is possible that anti-PD1 therapy affects the local tumor-microenvironment which may impact tumor growth and invasiveness. Therefore, to examine whether the unresponsiveness of OH-BBN induced Hgf-Cdk4^R24C^ tumors to anti-PD1 therapy was due to a progression-prone microenvironment and stroma, subcutaneously inoculated 74/7 tumors (growing on Hgf-Cdk4^R24C^ mice) were treated with anti-PD1 or CpG 1668 (Fig 6B). While anti-PD1 therapy did not impact tumor growth nor the well-being of the animals, subcutaneous tumor growth in Hgf-Cdk4^R24C^ mice was significantly reduced in CpG 1688 treated animals at day 22 (Fig 6D) (which was the latest time-point when all animals were still alive). When survival (Fig 6E) (Median survival: Female untreated: 31 days; Male untreated: 31 days; Female anti-PD1: 34 days; Male anti-PD1:29.5 days, Female CpG: not reached; Male CpG: not reached) and tumor size (Fig 6F–6K) were analyzed separately for each sex, a clear response of increased survival benefit (p = 0.05) (Fig 6E) and decreased tumor growth in CpG 1688 treated females (p = 0.026) (Fig 6K), but not males, was observed.

## Discussion

Recreating urothelial tumors in mouse models has been challenging. Gene expression signatures are not often consistent between transgenic models and human tumors, engraftment models do not develop relevant microenvironment, and carcinogen exposed models commonly do not reach the MIBC stage [18] or are not analyzed at that stage. Herein, we have induced urothelial cancer in transgenic Hgf-Cdk4^R24C^ mice whose germline genetic alterations lead to susceptibility towards development of tumors of epithelial origin spontaneously or upon carcinogen or UV radiation exposure [49,50]. Patients with bladder cancer present urothelial cell cycle dysregulation including aberrant activity of CDK4 [43,44], while overexpression of HGF is known to be involved in early stages of carcinogenesis [63]. Excessive expression of HGF by stromal cells promotes the survival and invasion of tumor cells [64], but also the polarization of immune microenvironment cells towards immunosuppressive phenotypes [65,66]. Thus, herein we present a model that harbors both pro-oncogenic factors leading to a dysregulated cell-cycle as well as invasiveness, along with a broad mutational burden by the induction of the tumorigenesis by OH-BBN.

The Hgf-Cdk4^R24C^ mice developed NMIBC tumors that progressed to MIBC over time suggesting that this model reflects a progressive rather than relapsing NMIBC. Additionally, the histopathology of Hgf-Cdk4^R24C^ bladder tumors was of urothelial carcinoma with squamous differentiation, which in humans is associated with progression, chemotherapy resistance and possibly disease progression after anti-PD1 therapy [67–71]. Also, the faster progression and shorter survival of Hgf-Cdk4^R24C^ mice compared to Cdk4^R24C^ with OH-BBN induced urothelial tumors indicates that the co-occurrence of both *Hgf* overexpression and overactivation of *Cdk4 (R24C)* led to a more progressive tumor phenotype.

Moreover, we observed a lack of metastases or tumor development in other sites than the bladder which were previously recorded in studies while using Hgf-Cdk4^R24C^ mice [49–51]. It is possible that this is a cancer specific property, as multiple metastases arise in very advanced stages in human patients usually after surgical removal of the bladder [72] or that circulating tumor cells did not evolve into tumor metastases due to the necessary humane endpoint reached when the animals reached the muscle invasive bladder tumor stage. We could not examine the metastatic potential, in relation to patients who present metastatic tumor growth after surgical removal of the bladder, due to lack of surgical measures as well as due to ethical constraints. Moreover, we attribute the lack of spontaneous tumors to the age of the mice used in our study (up to 7.5 months old), while it has been reported that mice of the Hgf-Cdk4^R24C^ mice develop tumors spontaneously after the average age of 8 months [52].

In this study, we generated and characterized six cell lines from single cell colonies of Hgf-Cdk4^R24C^ urothelial tumors, a strategy that might impact intratumoral heterogeneity and therefore limited tumor growth upon engraftment *in vivo*, as previously shown [73]. Indeed, five of six cell lines did not grow *in vivo*. Those were IFNγ responsive *in vitro*, a quality that is required of tumor cells for recognition and elimination by CD8 T cells. This could explain why 74/7, which did not respond to IFNγ by expression of immune recognition receptors, was the only cell line to form tumors upon engraftment.

The presence of genetically heterogeneous urothelial tumor foci was recently highlighted [74], while it became apparent that such heterogeneous tumor compartments are usually under-represented in patient tissue microarray biopsies [75,76]. Molecular subtypes are investigated by bulk sequencing on such biopsies, which probably limits the examination of intratumoral subtype heterogeneity. Recently, intratumoral heterogeneity in human urothelial bladder cancer was identified as clonal variety by DNA seq, where subclones of invasion were found in samples of progression, adding to the genetic mosaic theory in cancer [77]. In our study, the OH-BBN induced tumors displayed multifocal and heterogeneous histopathology with concomitant CIS similar to human bladder cancer [74–76]. To model a setup where this would be analyzed in a deeper level, we used single-cell RNAseq to decipher the transcriptomic heterogeneity of Hgf-Cdk4^R24C^ bladder tumor cells. Data analysis revealed distinct tumor and cancer stem cell-like cell clusters with different molecular subtype expression patterns in carcinogen induced tumors, contrary to homogeneous MB49 tumors. Our results altogether indicate that OH-BBN induced tumors in Hgf-Cdk4^R24C^ mice recapitulate human urothelial cancer induction, progression and heterogeneity. Moreover, we suggest that single cell analyses of clinical bladder cancer samples may shed light on whether coexisting heterogeneous tumor cell populations have impact on prognosis and/or therapy outcomes.

Despite the technical limitation of the PEA due to the lack of a reference protein to enable exact protein level comparison between mouse strains, proteomic analysis of liquid biopsies revealed that the novel mouse model has distinct profile from commonly used models, therefore suggesting an alternative shaping of tumor macro- and microenvironment in this strain. Repeated urine sampling of the transgenic strain over-time enabled us to capture the proteomic changes during early carcinogenesis, tumor establishment and at the MIBC stage. Those changes suggest that the tumor microenvironment was somewhat different between sexes, similar to humans [11] and that during carcinogenesis pre-tumoral urine proteomic alterations can be detected by highly sensitive PEA analysis.

Up to 75% of locally advanced MIBC or metastatic bladder cancer patients do not respond to anti-PD1 therapy [78,79]. This could be probably due to established immunosuppression in late stages that is beyond inversion or due to specific subtype related responses to immunotherapy as recently highlighted in a suggested classification system [80]. Despite the lack of a stratification method with high molecular precision in the clinic, anti-PD1 therapy is being tested in clinical trials to prevent recurrence and progression of high-risk NMIBC after tumor resection, as well as in conjunction with BCG therapy in a pre-surgical setting [81]. Recently, the FDA approved the use of anti-PD1 for BCG-unresponsive, high-risk NMIBC, after the therapy achieved responses in almost a third of patients [5]. In Hgf-Cdk4^R24C^ mice with OH-BBN induced tumors monotherapy with an anti-PD1 blocking antibody did not prevent the progression of non-invasive tumors. It is however plausible that tumor resection and combination therapies might be the key to achieve complete tumor regression for progressive disease or that the model could be a useful tool to further study mechanisms of immunotherapy resistance, since a recent study showed that anti-PD1 therapy of OH-BBN induced MIBC tumors in wild type C57BL/6 mice had a therapeutic effect [82]. Similar to OH-BBN induced bladder tumors, subcutaneous 74/7 tumors in Hgf-Cdk4^R24C^ mice did not respond to anti-PD1 monotherapy *in vivo* at a dose of 2–2.5 mg/kg. It is plausible that a higher dose anti-PD1 could achieve anti-tumor responses as a peritumoral dose of around 1.2–1.5 mg/kg did not impact MB49 tumor growth while an IV administration of 4–5 mg/kg was curative [83].

Interestingly, peritumoral CpG 1688 monotherapy significantly reduced tumor growth in female animals, while male mice did not respond to this therapy. The choice of this therapy was based on previous noted therapeutic impact on MB49 subcutaneous tumors inoculated in the C57BL/6 strain [15,83]. The systemic administration of CpG ODNs in oncology based clinical trials limited their potential, as CpG ODN infusion induced systemic cytokine release and narrowed the therapeutic window [84,85]. Therefore the administration route is key for success, as well as the presence of tumor antigens along with the adjuvant [86]. The use of these adjuvants as local immune-modulators in the neo-adjuvant setting in clinical trials is supported by publications [87]. Herein, we used peritumoral administration of CpG 1668 *in vivo* and observed a successful reduction of growth of 74/7 tumor graft in female hosts. Recently, a study elegantly showed that innate immune responses are more potent in females compared to males [12]. This suggests that in our study TLR stimulation by CpG 1688 possibly triggered a more potent anti-tumor response in female mice with subcutaneous 74/7 tumors. This is of particular interest as to date checkpoint blockade immunotherapy seems to yield a higher survival benefit for male rather than female patients, indicating that different therapeutic approaches should be considered among sexes [11,88,89]. This would require not only equal stratification of men and women to clinical trials, but also the use of pre-clinical models that are appropriate for drug target discovery and evaluation in terms of sex biology.

In conclusion the novel Hgf-Cdk4^R24C^ autochthonous urothelial cancer model and cell lines should be utilized to further understand the immuno-biology implications of tumor subtype and biological sex on therapeutic immune modulation in bladder cancer.

## Supporting information

S1 Fig Age related immune infiltration in Hgf-Cdk4^R24C^ carcinogen exposed bladders. (A) Summarized scoring of inflammation degree in bladder tissue at the 10-week endpoint of adult (10–14 wo) OH-BBN exposed (n = 7) and weaned (4–6 wo) OH-BBN exposed (n = 8) Hgf-Cdk4^R24C^ female mice. The heatmap scale indicates the fraction animals in each group that presented each inflammation level. Representative images of hematoxylin-eosin stained bladder sections from: (B) healthy female, (C) adult OH-BBN exposed and (D) weaned OH-BBN exposed Hgf-Cdk4^R24C^ female mice. Scale bars indicate 50 μm.
(TIF)

S2 Fig Significantly altered proteins (Mann Whitney, p<0.05) at NMIBC 10-week endpoint and MIBC endpoint in male and female Hgf-Cdk4^R24C^ OH-BBN induced mice and healthy controls. (A) Serum 10-week endpoint, (B) Urine 10-week endpoint, (C) Serum MIBC endpoint, (D) Urine MIBC endpoint. (E) Intersection illustration of the numbers of significantly upregulated and overlapping proteins between male and female mice at all time points (from GLS analysis).
(TIF)

S3 Fig Verification of Cdk4 (R24C) mutation presence and doubling time analysis of new cell lines. (A) The results of PCR-based DNA amplification of the Cdk4 gene region and presence of the R24C mutation as determined by cleavage by HINDIII endonuclease are shown. The MB49 cell line was used as negative control for the presence of R24C mutation, which was present only in Hgf-Cdk4^R24C^ derived cell lines. (B)The doubling time of the new cell lines, determined by the function y = a2Tx-b (a, b- parameters, T- doubling time), was in the range of 14.3h-20h, except for the cell line 73/2 which had doubling time of 31.2h.
(TIF)

S1 Table List of primer pairs used in qRT-PCR. (DOCX)

S2 Table List of antibodies used for flow cytometry staining. (DOCX)

S1 File Differential expression of urothelial cell clusters analyzed by single cell RNA sequencing. (TXT)

S2 File KEGG enrichment of urine proteomic analysis at day 24 in female Hgf-Cdk4^R24C^ mice. (CSV)

S3 File KEGG enrichment of urine proteomic analysis at day 24 in male Hgf-Cdk4^R24C^ mice. (CSV)

S4 File KEGG enrichment of urine proteomic analysis upon progression to MIBC in female Hgf-Cdk4^R24C^ mice. (CSV)

S5 File KEGG enrichment of urine proteomic analysis upon progression to MIBC in male Hgf-Cdk4^R24C^ mice. (CSV)

S6 File NPX values of serum Olink proteomic analysis. (CSV)

S7 File NPX values of urine Olink proteomic analysis. (CSV)

S1 Raw images Raw gel image for S3 Fig. Captured on Gel Doc EZ Gel Documentation System (Bio Rad).
(PDF)
