# Correction: Single-cell RNAseq and longitudinal proteomic analysis of a novel semi-spontaneous urothelial cancer model reveals tumor cell heterogeneity and pretumoral urine protein alterations

**DOI:** 10.1371/journal.pone.0304890

**Published:** 2024-05-31

**Authors:** Iliana K. Kerzeli, Martin Lord, Milena Doroszko, Ramy Elgendy, Aikaterini Chourlia, Ivan Stepanek, Elinor Larsson, Luuk van Hooren, Sven Nelander, Per-Uno Malmstrom, Anca Dragomir, Ulrika Segersten, Sara M. Mangsbo

In Fig 4, the panels A and B are duplicated. Please see the correct version of Fig 4 here.

Orthotopic MB49 model (day 5), carcinogen induced wild type female C57BL/6 (10-week endpoint) and carcinogen induced male and female Hgf-Cdk4^R24C^ (10-week and MIBC endpoint). Significantly altered serum proteins in (A) MB49 orthotopic model (n = 6), (B) Carcinogen induced female C57BL/6 mice (n = 6), (C) 10-week endpoint Hgf-Cdk4^R24C^ mice (n = 13), (D) MIBC endpoint Hgf-Cdk4^R24C^ mice (n = 8) compared to respective healthy controls (n = 6–10). Levels of significantly changed protein levels in the urine samples of (E) MB49 orthotopic model (n = 4), (F) Carcinogen induced female C57BL/6 mice (n = 6), (G) 10-week endpoint Hgf-Cdk4^R24C^ mice (n = 18), (H) MIBC endpoint Hgf-Cdk4^R24C^ mice (n = 8) compared to respective healthy controls (n = 4–13). Fold change for each protein was calculated as 2^(mean NPX cancer—mean NPX healthy control)^. Colored circles indicate significance level of p<0.05; red for increased proteins and blue for decreased proteins for cancer model compared to healthy controls, grey for proteins below the significance level.

Moreover, there are errors in the captions of Fig 3 and 5. In Fig 3, the legend “D” should have been “E”. Then, in Fig 5, the legend “E” is missing so the legend that appears as “F” should be “E”, the “H” should be “G”, the “G” should be “f” and the “I” should be “H”. Please see the complete, correct Figs 3 and 5 captions here.

**Fig 3:**
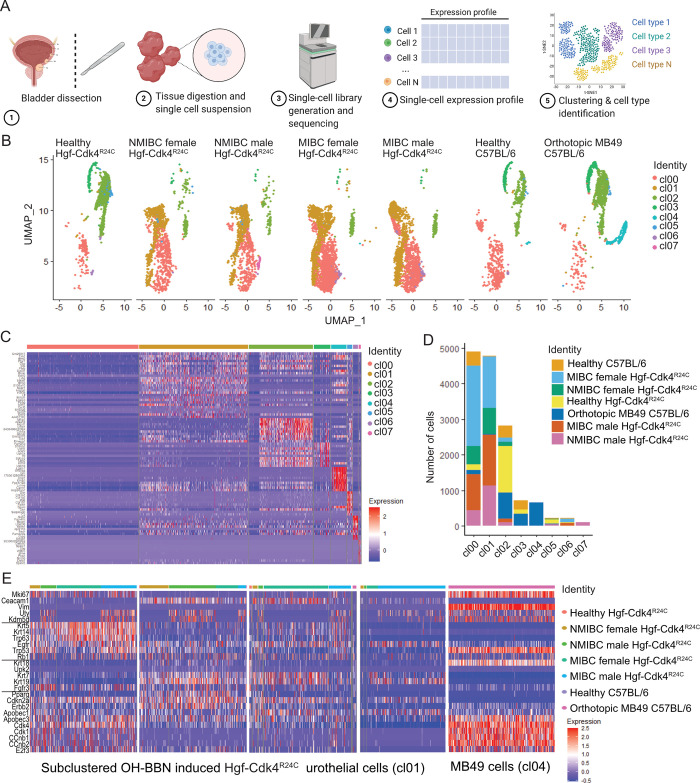
Single cell transcriptomic analysis of the tumor bearing and healthy bladders. (A) Workflow of the single-cell RNAseq, and (B) UMAP projection of all urinary epithelium cell clusters (cl00-cl07) in all of the samples analyzed by single-cell RNAseq. (C) Transcriptomic heterogeneity of the identified urothelial cell clusters and (D) number of cells from each cluster in each bladder analyzed for Hgf-Cdk4R24C or C57BL/6 mice. Out of all cell clusters in Hgf-Cdk4R24C and MB49 tumor bearing bladders (B, C, D), cl01 which is present only in OH-BBN induced Hgf-Cdk4R24C bladders and cl04 which is present only in MB49 tumor bearing bladders, were further subsetted/subclustered and the expression of (E) tumor or cancer stem cell marker genes, as well as molecular subtype relevant genes is visualized for each model.

**Fig 4:**
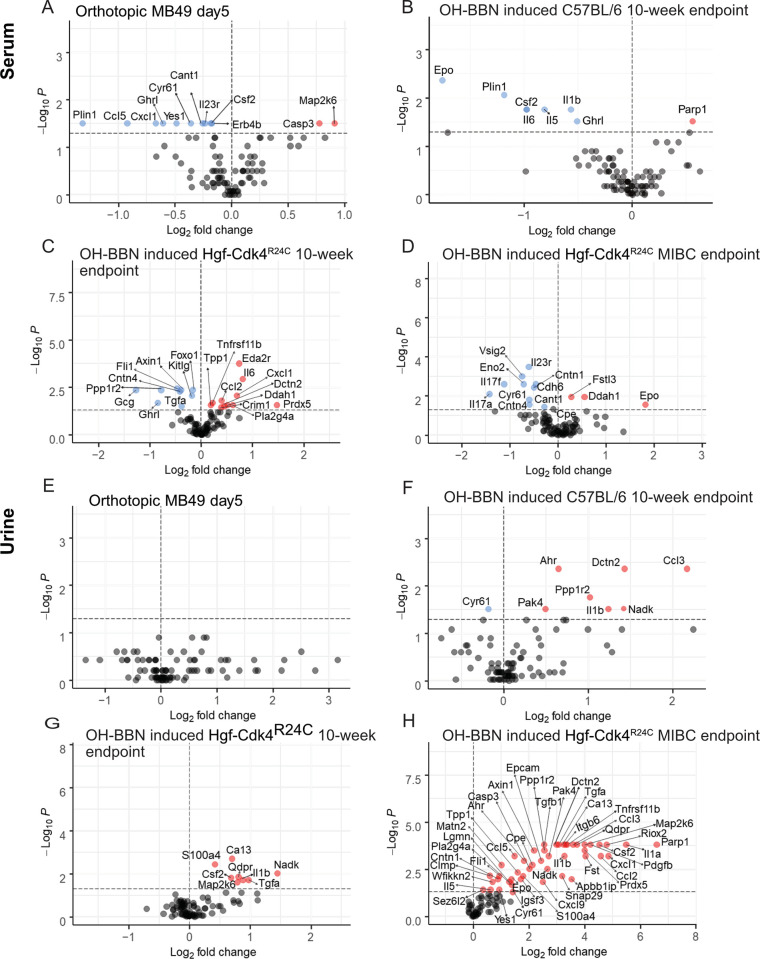
Protein signatures in serum and urine in three murine urothelial cancer models.

**Fig 5:**
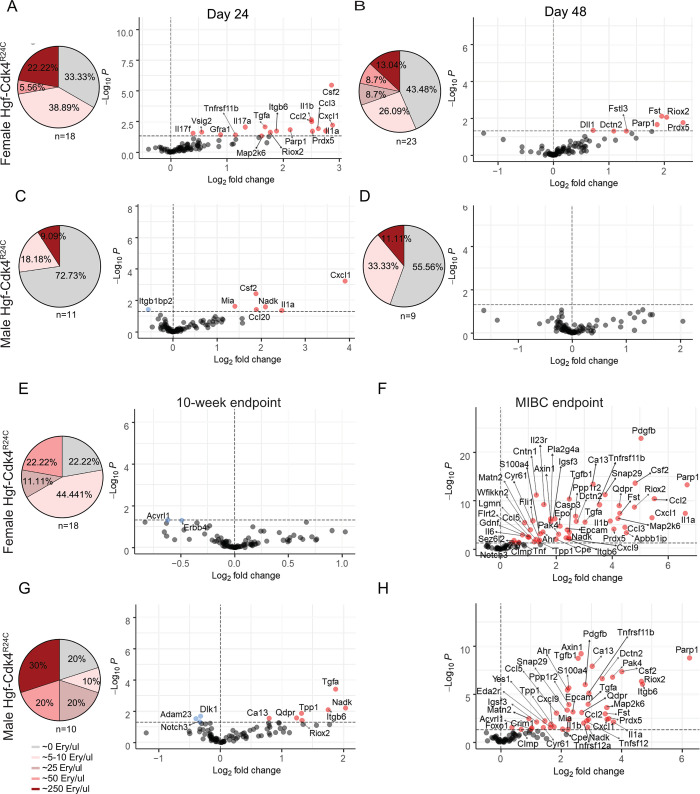
Microhematuria levels and sex specific protein alterations in urine over time in Hgf-Cdk4R24C OH-BBN induced mice depict events of carcinogenesis, early immune response and tumor progression. From day 24 until the 10-week endpoint a percentage of animals presented none, low or high levels of microhematuria, expressed as erythrocytes/μl. At the MIBC endpoint gross hematuria was noted and therefore no microhematuria measurements were performed. Day 24 [(A) Females: n = 11 cancer, n = 3 healthy. (C) Males: n = 7 cancer, n = 3 healthy], day 48 [(B) Females: n = 11 cancer, n = 3 healthy. (D) Males: n = 6 cancer, n = 3 healthy], 10-week endpoint [(E) Females: n = 11 cancer, n = 6 healthy. (G) Males: n = 7 cancer, n = 7 healthy], MIBC endpoint [(F) Females: n = 5 cancer, n = 4 healthy. (H) Males: n = 3 cancer, n = 4 healthy]. Repeated measurements were analyzed by a generalized least square (GLS) to model the effect of gender on the disease at a specific time point. Fold change was calculated as 2(mean NPX cancer—mean NPX healthy control). Colored circles indicate significance level of p<0.05; red for increased proteins and blue for decreased proteins for each time point compared to healthy controls, grey circles show proteins below the significance level.
