# Supplementary figures and images for: Single-cell RNAseq and longitudinal proteomic analysis of a novel semi-spontaneous urothelial cancer model reveals tumor cell heterogeneity and pretumoral urine protein alterations
Source: PLoS One. 2021 Jul 7;16(7):e0253178. doi: 10.1371/journal.pone.0253178 (PMC8262791; doi:10.1371/journal.pone.0253178)

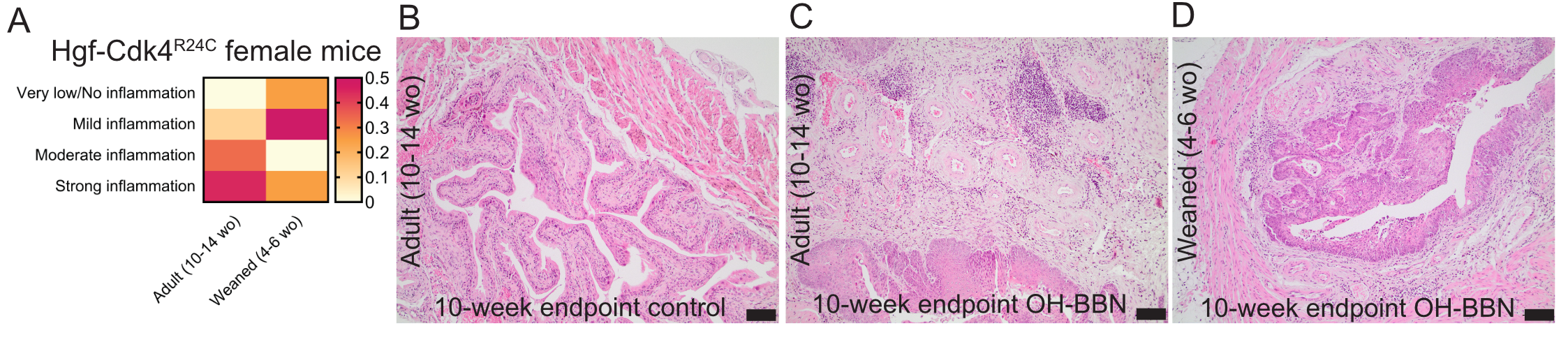

Supplement: S1 Fig — (A) Summarized scoring of inflammation degree in bladder tissue at the 10-week endpoint of adult (10–14 wo) OH-BBN exposed (n = 7) and weaned (4–6 wo) OH-BBN exposed (n = 8) Hgf-Cdk4R24C female mice. The heatmap scale indicates the fraction animals in each group that presented each inflammation level. Representative images of hematoxylin-eosin stained bladder sections from: (B) healthy female, (C) adult OH-BBN exposed and (D) weaned OH-BBN exposed Hgf-Cdk4R24C female mice. Scale bars indicate 50 μm. (TIF) [file pone.0253178.s001.tif]

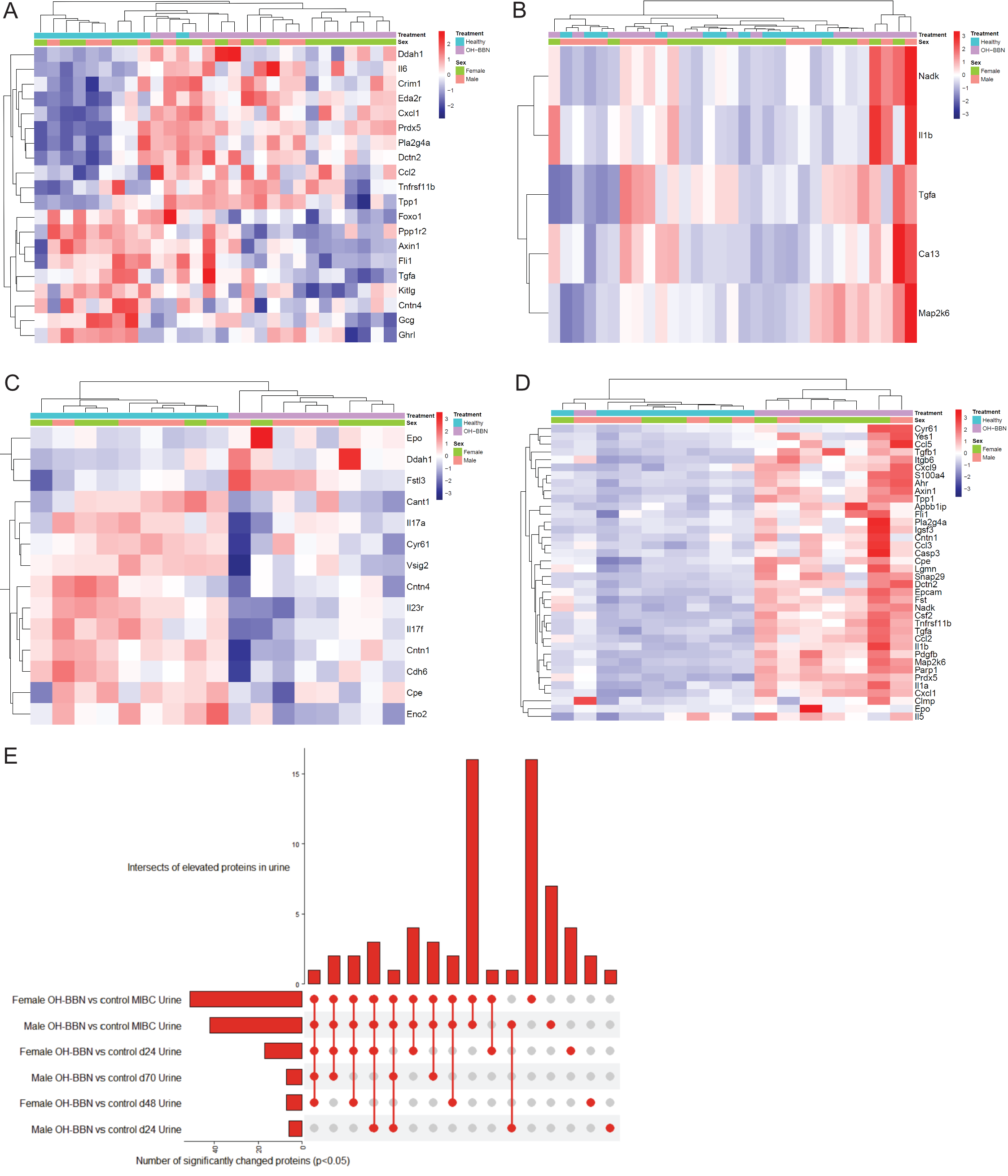

Supplement: S2 Fig — (A) Serum 10-week endpoint, (B) Urine 10-week endpoint, (C) Serum MIBC endpoint, (D) Urine MIBC endpoint. (E) Intersection illustration of the numbers of significantly upregulated and overlapping proteins between male and female mice at all time points (from GLS analysis). (TIF) [file pone.0253178.s002.tif]

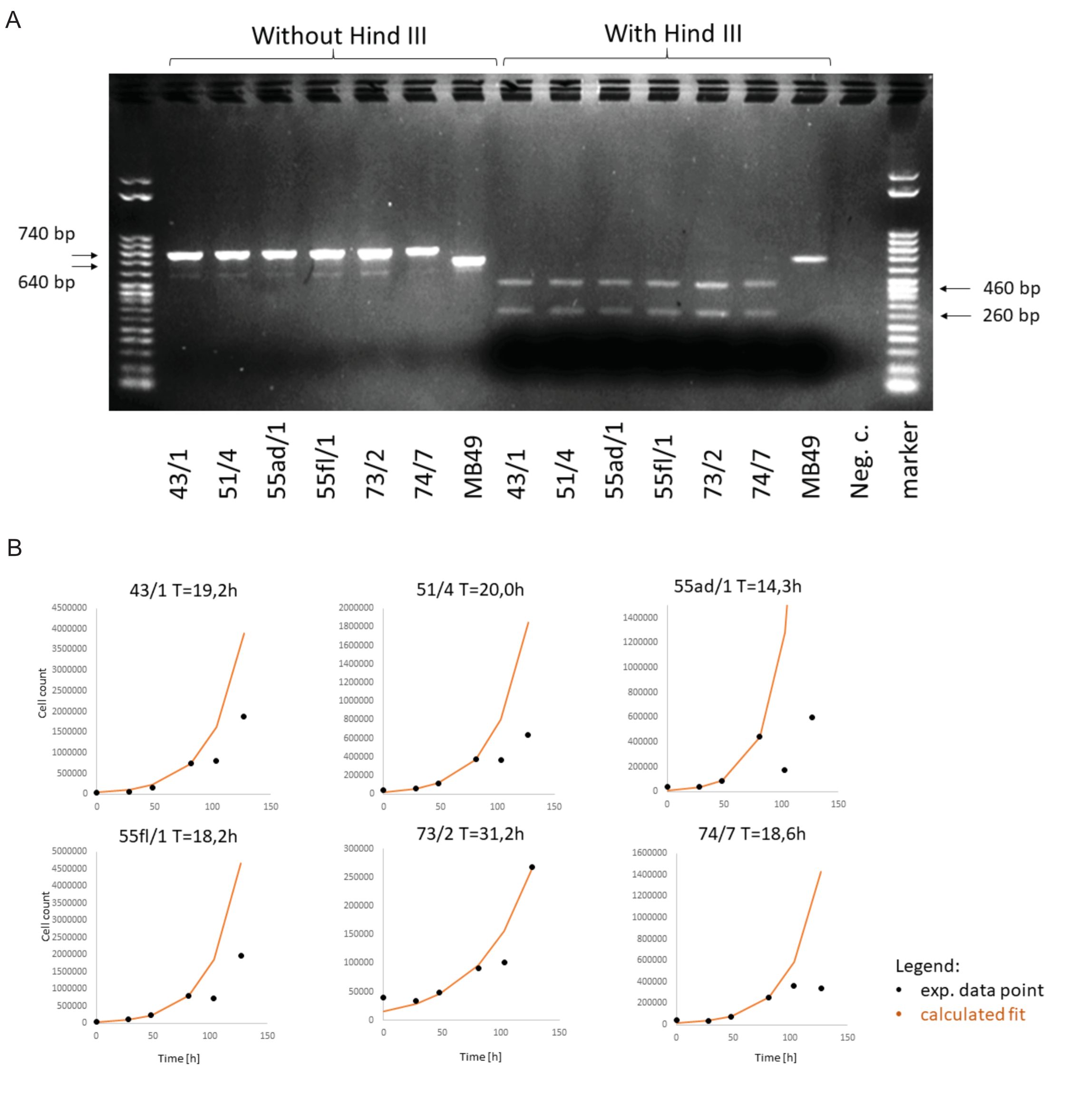

Supplement: S3 Fig — (A) The results of PCR-based DNA amplification of the Cdk4 gene region and presence of the R24C mutation as determined by cleavage by HINDIII endonuclease are shown. The MB49 cell line was used as negative control for the presence of R24C mutation, which was present only in Hgf-Cdk4R24C derived cell lines. (B)The doubling time of the new cell lines, determined by the function y = a2Tx-b (a, b- parameters, T- doubling time), was in the range of 14.3h-20h, except for the cell line 73/2 which had doubling time of 31.2h. (TIF) [file pone.0253178.s003.tif]
