# Supplementary material for: Single-cell RNAseq and longitudinal proteomic analysis of a novel semi-spontaneous urothelial cancer model reveals tumor cell heterogeneity and pretumoral urine protein alterations
Source: PLoS One. 2021 Jul 7;16(7):e0253178. doi: 10.1371/journal.pone.0253178 (PMC8262791; doi:10.1371/journal.pone.0253178)
Supplement: S1 Table — (DOCX) [file pone.0253178.s004.docx]

| Mouse gene | Forward primer | Reverse primer |
| --- | --- | --- |
| Krt7 | GCGGAGATGAACCGCTCTAT | CTAACTTGGCACGCTGGTTC |
| Actin-beta | CGCAGCCACTGTCGAGTC | GTCATCCATGGCGAACTGGT |
| Krt5 | TGAGGAGCTGCAACAGACAG | AGGTTGGCACACTGCTTCTT |
| Krt20 | AGTTCGAGAGACAGAGTCAAAC | TAGGTTGCGCTCCAGAGACT |
| Krt14 | GTGAAGACAAGGCTGGAGCA | CATGACCTTGGTGCGGATCT |
| Trp63 | ACCCTTACATCCAGCGTTTCAT | CTCGATGGGCTGTACTGAGC |
| Krt18 | AGAGACTGGGGCCACTACTT | TGGCTAGTTCTGTCTCATACTTGA |
| Cdh1 | CAGCCGGTCTTTGAGGGATT | GGTAACTCTCTCGGTCCAGC |
| Cdh2 | CGCAGTCTTACCGAAGGATGT | CCTTGAAATCTGCTGGCTCG |
| Des | GTGCATGAAGAGGAGATCCGT | ATGTTCTTAGCCGCGATGGT |
| Myh11 | CGACACAGCCTACAGAAGCA | TTTTCCAGCTCCCCCGTGAT |
| Vim | AGAGAGGAAGCCGAAAGCAC | TGGATCTCTTCATCGTGCAGT |
| Pdgfra | GGAACCTCAGAGAGAATCGGC | CATAGCTCCTGAGACCCGCTG |
| Col1a2 | TACCTGGATGAGGAGACGGG | GGTGCAATGTCAAGGAACGG |
| Upk2 | ACCAGGAACCAAATACTACATATCC | AGAGCCCTTCTCATTTGCGG |
| Tagln | GAAGGTGCCTGAGAACCCAC | GCTCCTGGGCTTTCTTCATAAAC |
