# Supplementary material for: Single-cell RNAseq and longitudinal proteomic analysis of a novel semi-spontaneous urothelial cancer model reveals tumor cell heterogeneity and pretumoral urine protein alterations
Source: PLoS One. 2021 Jul 7;16(7):e0253178. doi: 10.1371/journal.pone.0253178 (PMC8262791; doi:10.1371/journal.pone.0253178)
Supplement: S2 Table — (DOCX) [file pone.0253178.s005.docx]

| Target | Clone | Fluorophore | Cat no | Manufacturer |
| --- | --- | --- | --- | --- |
| H-2Kb | AF6-88.5 | PE | 116507 | Biolegend |
| I-a | M5.114.15.2 | APC | 107614 | Biolegend |
| CD80 | 16-10A1 | BV421 | 104725 | Biolegend |
| H-2Db | KH95 | PE | 111507 | Biolegend |
| CD54 (Icam-1) | YN1/1.7.4 | APC | 116119 | Biolegend |
| CD274(PD-L1) | 10F.962 | BV421 | 124315 | Biolegend |
| FASL | MFL3 | PE | 106605 | Biolegend |
| CD140a (Pdgfra) | APA5 | APC | 135907 | Biolegend |
| CD324(E-cadherin) | DECMA-1 | BV421 | 147319 | Biolegend |
