# Supplementary material for: Single-cell RNAseq and longitudinal proteomic analysis of a novel semi-spontaneous urothelial cancer model reveals tumor cell heterogeneity and pretumoral urine protein alterations
Source: PLoS One. 2021 Jul 7;16(7):e0253178. doi: 10.1371/journal.pone.0253178 (PMC8262791; doi:10.1371/journal.pone.0253178)
Supplement: S1 Raw images — Captured on Gel Doc EZ Gel Documentation System (Bio Rad). (PDF) [file pone.0253178.s013.pdf]

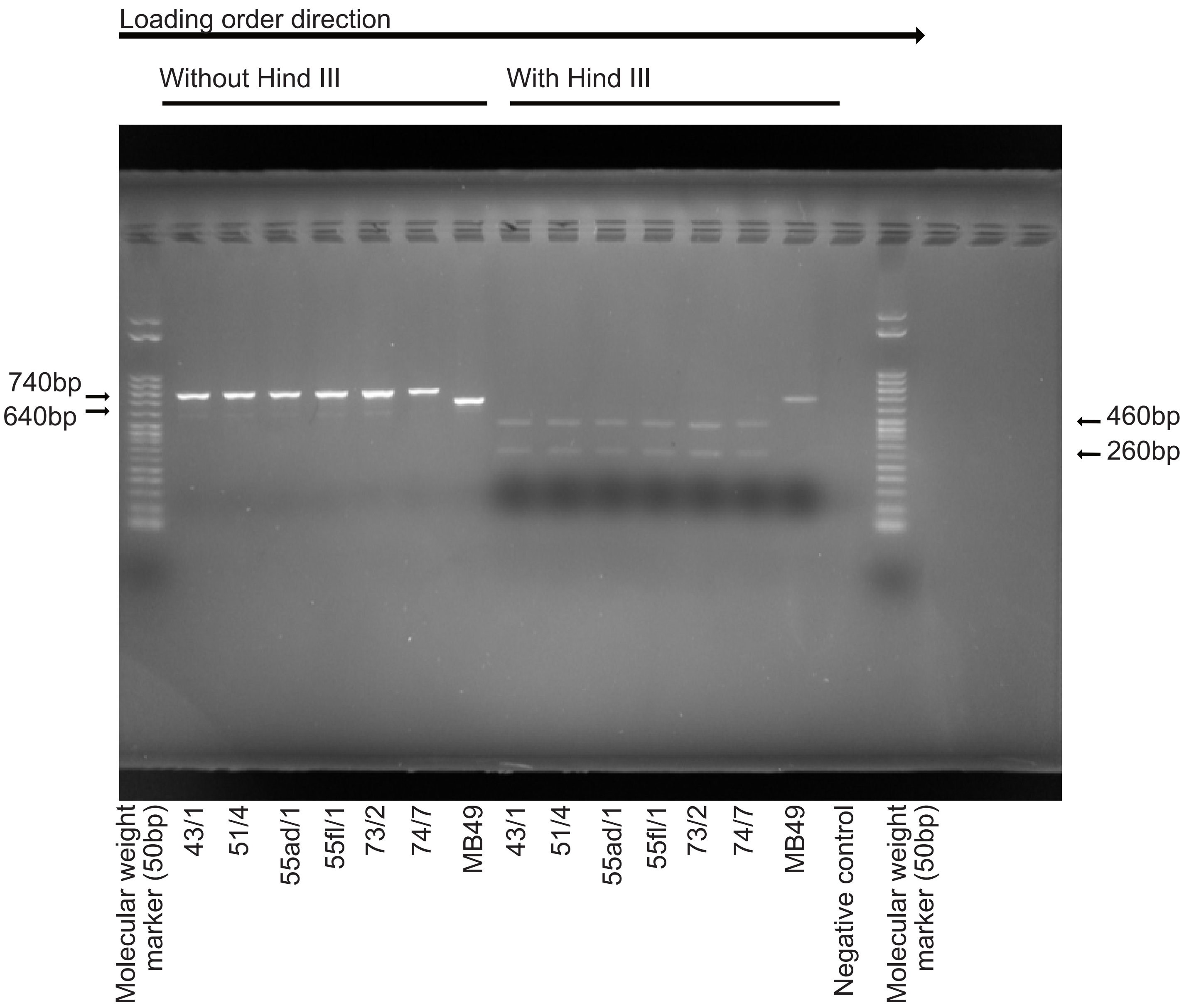

Raw gel image for S3 Fig. Captured on Gel Doc EZ Gel Documentation System (Bio Rad).
